# Eye opening differentially modulates inhibitory synaptic transmission in the developing visual cortex

**DOI:** 10.7554/eLife.32337

**Published:** 2017-12-11

**Authors:** Wuqiang Guan, Jun-Wei Cao, Lin-Yun Liu, Zhi-Hao Zhao, Yinghui Fu, Yong-Chun Yu

**Affiliations:** 1 Jing’an District Center Hospital of Shanghai, Institutes of Brain Science, State Key Laboratory of Medical Neurobiology and Collaborative Innovation Center for Brain Science, Fudan UniversityShanghaiChina; University of FreiburgUnited States

**Keywords:** eye opening, synaptic transmission, somatostatin-expressing interneurons, fast-spiking interneurons, development, Mouse

## Abstract

Eye opening, a natural and timed event during animal development, influences cortical circuit assembly and maturation; yet, little is known about its precise effect on inhibitory synaptic connections. Here, we show that coinciding with eye opening, the strength of unitary inhibitory postsynaptic currents (uIPSCs) from somatostatin-expressing interneurons (Sst-INs) to nearby excitatory neurons, but not interneurons, sharply decreases in layer 2/3 of the mouse visual cortex. In contrast, the strength of uIPSCs from fast-spiking interneurons (FS-INs) to excitatory neurons significantly increases during eye opening. More importantly, these developmental changes can be prevented by dark rearing or binocular lid suture, and reproduced by the artificial opening of sutured lids. Mechanistically, this differential maturation of synaptic transmission is accompanied by a significant change in the postsynaptic quantal size. Together, our study reveals a differential regulation in GABAergic circuits in the cortex driven by eye opening may be crucial for cortical maturation and function.

## Introduction

During neocortical development, sensory experience critically inﬂuences neuronal connectivity and synaptic transmission (Katz and Shatz, 1996). In the visual system, maturation of the visual cortex depends on visual afferent activity (Hensch, 2005; Hofer et al., 2009; Pecka et al., 2014). The initial visual inputs experienced through closed eyelids are imprecise and diffuse, and followed by patterned visual inputs after eye opening. In general, experience-dependent maturation of the visual cortex is a gradual process, but it accelerates soon after eye opening when the intensity and frequency of afferent visual activity suddenly and rapidly increase (Lu and Constantine-Paton, 2004). Eye opening in rodents typically occurs over a short period of one to two days (Gandhi et al., 2005). When experimentally synchronized, eye opening drives a rapid series of changes in neuronal activity, protein trafficking, synaptogenesis, synaptic receptor composition, and synaptic transmission and plasticity (Lu and Constantine-Paton, 2004; Yoshii et al., 2003; Zhao et al., 2006). Moreover, eye opening affects neuronal circuit development in the ascending retinothalamo-cortical pathway at every level: the retina (Feller, 2003; Tian and Copenhagen, 2003), superior colliculus (SC) (Zhao et al., 2006), lateral geniculate nucleus (LGN) (Hooks and Chen, 2006; Levitt et al., 2001), and visual cortex (Hoy and Niell, 2015; Ko et al., 2013; Pecka et al., 2014). For example, the probability and strength of excitatory connections between layer 2/3 pyramidal cells (PCs) in the visual cortex significantly increased after eye opening, and these changes were prevented by dark rearing (Ishikawa et al., 2014). Although previous study reported that orientation tuning preferences of fast-spiking interneurons (FS-INs) were dependent on normal visual experience after eye opening (Kuhlman et al., 2011), whether eye opening shapes inhibitory synaptic transmission in the neocortex during development remains unclear.

In the mature neocortex, GABAergic INs can be categorized into three main populations based on the expression of the calcium-binding protein parvalbumin (PV), the neuropeptide somatostatin (Sst), and the ionotropic serotonin receptor (Lee et al., 2010). Sst-expressing INs (Sst-INs), comprising approximately 20 ~ 30% of all neocortical INs, are among the most prominent GABAergic IN subtypes in the neocortex (Lee et al., 2010; Pfeffer et al., 2013). Sst expression in the superficial neocortex has typically been associated with Martinotti cells (MCs)—GABAergic INs with ascending axons that arborize in cortical layer one and spread horizontally to neighboring columns (Fino and Yuste, 2011; Ma et al., 2006). A large fraction of Sst-INs preferentially target distal dendrites of PCs (Di Cristo et al., 2004). In addition to providing lateral inhibition to local PC networks, cortical Sst-INs also precisely control the efficacy and plasticity of glutamatergic inputs by regulating postsynaptic spine Ca^2+^ signals, synaptic dynamics, dendritic spike bursts, and transformation of dendritic inputs (Chiu et al., 2013; Higley, 2014; Lovett-Barron et al., 2012). Furthermore, cortical Sst-INs not only innervate PCs but also frequently inhibit other cortical INs (disinhibition) (Pfeffer et al., 2013). PV-expressing FS-INs, which constitute ~40% of cortical GABAergic neurons, form powerful synapses onto the somatic and perisomatic compartments of PCs (Di Cristo et al., 2004). Although the emergence and maturation of connections from PV-INs to PCs and PV-INs have been reported previously (Lazarus and Huang, 2011); Pangratz-Fuehrer and Hestrin, 2011); Yang et al., 2014), the development of inhibitory synaptic transmission (including amplitude and connectivity) from Sst-INs to PCs and other IN subtypes remains largely unclear. In addition, accumulating evidence indicates that Sst-INs regulate learning-induced and experience-dependent cortical plasticity through feedforward as well as feedback inhibitions, both during early postnatal development and in adulthood (Bloodgood et al., 2013; Chen et al., 2015; Marques-Smith et al., 2016; Oh et al., 2016; Tuncdemir et al., 2016). There is also clear evidence suggesting that PV-INs regulate critical-period experience-dependent plasticity (Hensch, 2005; Krishnan et al., 2015). These observations raise important questions: Does eye opening play a role in the regulation of inhibitory synaptic transmission from Sst-INs and FS-INs to PCs or other types of INs, and if so, what is the synaptic mechanism underlying the regulation?

In this study, we demonstrate that eye opening rapidly weakens the inhibitory synaptic transmission from Sst-INs to PCs, whereas it increases inhibitory synaptic transmission from FS-INs to PCs. Moreover, we show that the maturation of inhibitory synaptic transmission is mediated by differential changes in the postsynaptic quantal size.

## Results

### Rapid weakening of synaptic transmission from Sst-INs onto PCs coincides with eye opening

To explicitly identify Sst-INs in the neocortex, we crossed the *Sst-IRES-Cre* mice with the loxP-flanked *Rosa26reporter-tdTomato* mice. Sst-INs in the resulting progeny expressed red fluorescent protein tdTomato in the brain, and this facilitated electrophysiological recordings of Sst-INs (Taniguchi et al., 2011). We focused on layer 2/3 of the primary visual cortex. Consistent with previous reports (Hu et al., 2013; Pfeffer et al., 2013), we observed that ~5.2% of tdTomato^+^ neurons expressed PV (5.2 ± 0.3%, nine slices from three mice, Figure 1—figure supplement 1B). Meanwhile, ~17.2% of tdTomato^+^ neurons showed the FS properties (36 out of 209, Figure 1—figure supplement 1C) (Hu et al., 2013; Jiang et al., 2015). Furthermore, FS tdTomato^+^ cells exhibited the distinctive basket cell morphology (4 out of 4, Figure 1—figure supplement 1C). These cells were omitted from further analysis. Non-FS tdTomato^+^ cells were further characterized by morphological properties, including ascending axonal arborizations with extensive branching in layer one and horizontal collaterals (19 out of 20, Figure 1—figure supplement 1A), consistent with those of Martinotti cells as previously described (Fino and Yuste, 2011).

**Figure 1.**
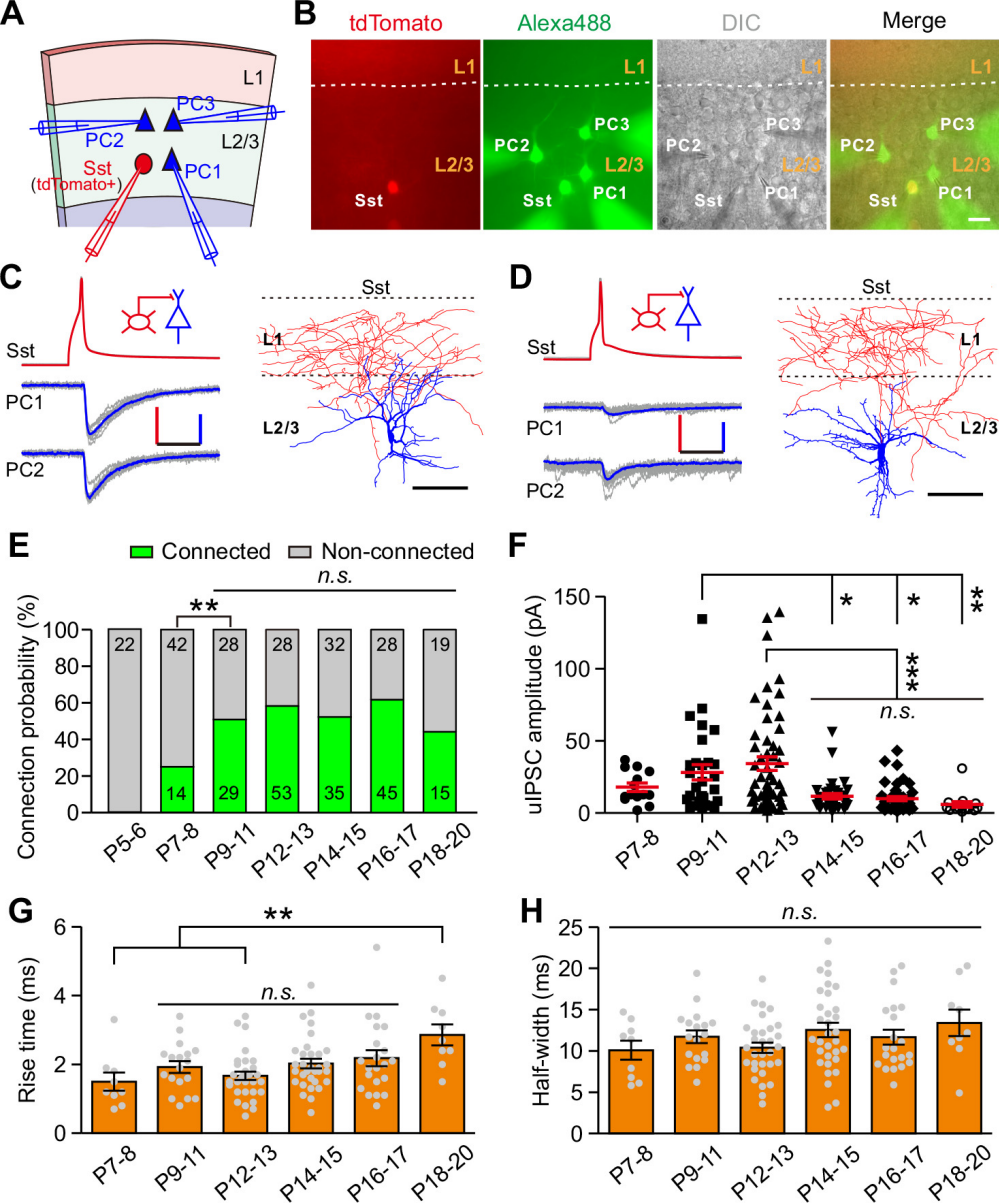
Development of synaptic transmission from Sst-INs onto PCs in layer 2/3 of the visual cortex. (**A**) Schema of a quadruple whole-cell recording from an Sst-IN (red) and three PCs (blue) in layer 2/3. (**B**) Representative fluorescent (tdTomato, Sst-INs; Alexa 488, recorded neurons), IR-DIC, and merged images of a quadruple recording of an Sst-IN and three PCs. The dashed lines indicate the border between layer 1 and layer 2/3. Scale bar, 20 μm. (**C**) *Left*, representative traces showing synaptic transmission from an Sst-IN to two PCs recorded at P11. The red and blue lines indicate the averaged traces. Scale bars: 50 mV (vertical, red), 25 pA (vertical, blue), and 20 ms (horizontal). *Right*, morphological reconstruction of the Sst-IN. Scale bar: 80 µm. (**D**) *Left*, representative traces showing synaptic transmission from an Sst-IN to two PCs recorded at P15. Scale bars: 50 mV (vertical, red), 25 pA (vertical, blue), and 20 ms (horizontal). *Right*, morphological reconstruction of the Sst-IN. Scale bar: 80 µm. (**E**) The probability of synaptic connection from Sst-INs to PCs at P5–20. Data label indicates the number of pairs in each group. A total of 391 pairs were recorded from 82 mice. (**F**) Quantification of the peak amplitude of uIPSCs from Sst-INs to PCs at different postnatal ages. (**G–H**) Quantification of the 10–90% rise time (**G**) and half-width (**H**) of uIPSCs at P7–20. Detailed statistical analysis, detailed data, and exact sample numbers are presented in Figure 1—source data 1. Error bars indicate mean ±SEM. *p<0.05; **p<0.01; ***p<0.001; *n.s.*, p>0.05. 10.7554/eLife.32337.007Figure 1—source data 1.Detailed statistical analysis, detailed data, exact sample numbers, and *p* values in Figure 1 and Figure 1—figure supplement 1–4.

To study synaptic transmission from Sst-INs to PCs, we performed triple or quadruple whole-cell patch-clamp recordings to record three or four cells in layer 2/3 simultaneously: a tdTomato^+^ Sst-IN and two or three nearby PCs whose cell bodies were within ~100 µm apart (Figure 1A and B). The PCs were identified by morphological characteristics (i.e., a large pyramidal shaped soma, basal and apical dendrites decorated with spines) and firing properties (Figure 1—figure supplement 3A and Table 1) (Lazarus and Huang, 2011; Schubert et al., 2001). Once all recordings were established, serial action potentials (at least 20 trials) were triggered in tdTomato^+^ Sst-IN, and the inward unitary inhibitory postsynaptic currents (uIPSCs) were measured in PCs (Figure 1C and D). To systematically study the development of synaptic transmission from Sst-INs to PCs (Sst-INs→PCs), we examined 391 Sst-IN→PC pairs at different developmental stages. We found that Sst-IN→PC connections emerged at postnatal day 7–8 (P7–8). The connection probability increased significantly from P7–8 to P9–11 (χ^2^ test, p=0.0046; Figure 1E) and remained largely comparable from P9 to P20 (χ^2^ test, p=0.149; Figure 1E). Interestingly, we observed a ~65% reduction in the peak amplitude of uIPSCs from P12–13 to P14–15 (P7–8, 17.9 ± 2.9 pA; P9–11, 28.3 ± 5.4 pA; P12–13, 34.3 ± 4.8 pA; P14–15, 11.6 ± 1.8 pA; P16–17, 10.0 ± 1.4 pA; P18–20, 6.0 ± 1.9 pA; one-way ANOVA, *F*_(5,185)_ *=* 8.788, p=1.7 × 10^−7^; Figure 1F). Notably, this dramatic change coincided with the natural eye opening of mice under standard living conditions (Figure 1—figure supplement 2), suggesting that the weakening of Sst-IN→PC uIPSCs may result from eye opening. Although the 10–90% rise time of uIPSCs at P18–20 was significantly longer than that at P7–8 and P12–13, the 10–90% rise time of uIPSCs did not significantly change at P9–17 (Figure 1G). Furthermore, the half-width of uIPSCs did not show any obvious change at P7–20 (Figure 1H). In addition, the total length and complexity of both apical and basal dendrites of PCs exhibited no significant difference between P12–13 and P14–15 mice (Figure 1—figure supplement 3B–I), suggesting that dendritic morphology of PCs remains unchanged during eye opening. Of note, the connection probability and strength of Sst-IN→PC synaptic transmission exhibited similar development properties when we used cesium-based and high Cl^-^ internal solution to record the postsynaptic currents (Figure 1—figure supplement 4).

**Figure 1—figure supplement 1.**
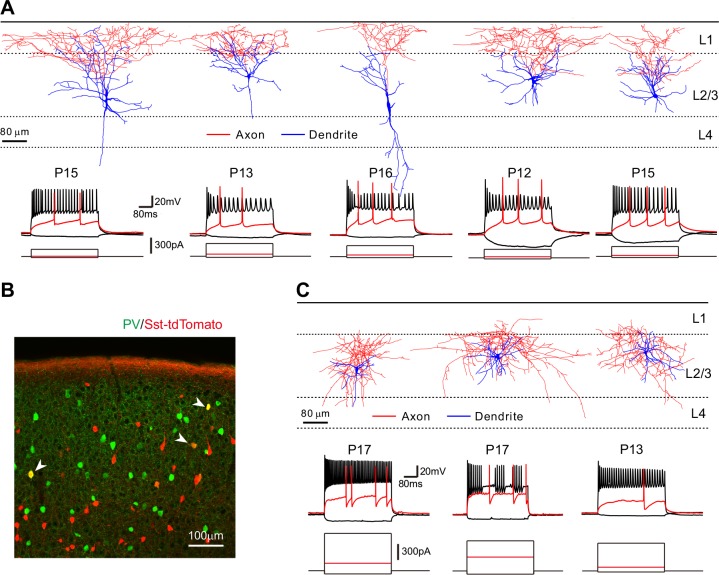
Morphological and electrophysiological properties of tdTomato^+^ neurons in *Sst-tdTomato* mice. (**A**) *Top panel*, the morphologies of reconstructed non-FS tdTomato^+^ neurons in layer 2/3 of visual cortex. *Bottom panel*, corresponding traces of voltage responses to 500 ms current pulse step injections recorded in the current-clamp mode. (**B**) Some tdTomato^+^ cells in *Sst-tdTomato* line expressed PV. Arrowheads indicated PV^+^/tdTomato^+^ cells. (**C**) *Top panel*, the morphologies of reconstructed fast-spiking tdTomato^+^ cells. These cells are basket-like interneurons, with dense axonal arborization in layer 2/3. *Bottom panel*, corresponding membrane voltage responses of cells to current injections.

**Figure 1—figure supplement 2.**
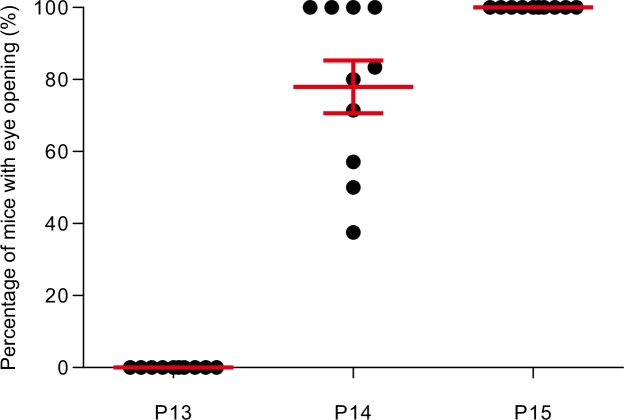
The timing of eye opening in mice. Quantification of mice undergoing eye opening in each litter (n = 10) from P13 to P15. There were no mice with opened eyes at P13. At P14, 77.9% ± 7.4% of mice had opened eyes; at P15, 100% of mice had opened eyes.

**Figure 1—figure supplement 3.**
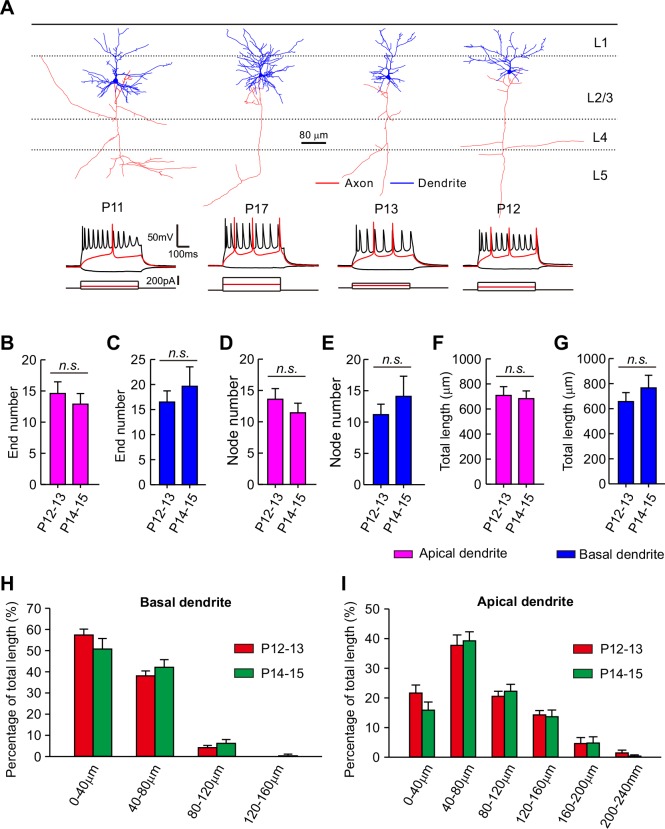
Dendritic morphology of PCs does not change significantly before and after eye opening. (**A**) The morphological identity of recorded PCs. *Top panel*, the morphologies of reconstructed PCs. *Bottom panel*, corresponding membrane responses of PCs to current injections. (**B–G**) Quantiﬁcation of the end number of apical dendrites (**B**), the end number of basal dendrites (**C**), the node number of apical dendrites (**D**), the node number of basal dendrites (**E**), the total length of apical dendrites (**F**), and the total length of basal dendrites (**G**) between P12–13 (before eye opening) and P14–15 (after eye opening). (**H–I**) Sholl analysis of dendritic length per 40 μm radial unit distance from the soma. Detailed statistical analysis, detailed data, and exact sample numbers are presented in **Figure 1—source data 1.** *n.s.*, p>0.05.

**Figure 1—figure supplement 4.**
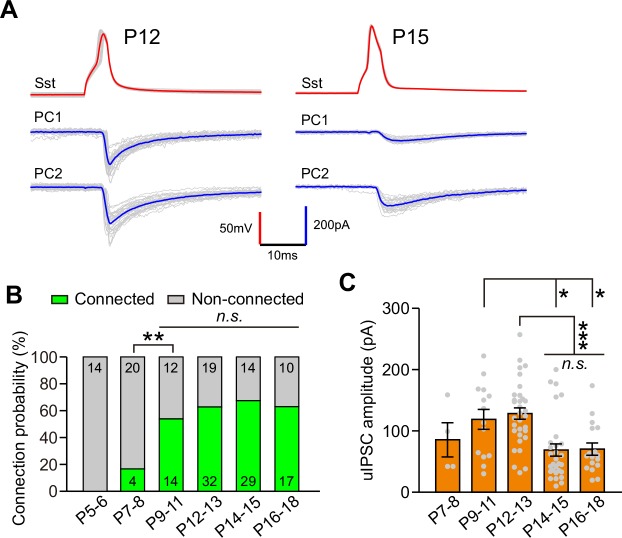
Synaptic responses from Sst-INs to PCs recorded with a cesium-based intracellular solution. (**A**) Representative traces showing synaptic transmission from Sst-INs to PCs recorded at P12 and P15, respectively. Postsynaptic responses were recorded with a cesium-based intracellular solution containing 60 mM Cl^-^. (**B**) Summary of connection probability. (**C**) Quantification of the peak amplitude of Sst-IN→PC uIPSCs. Detailed statistical analysis, detailed data and exact sample numbers are presented in Figure 1—source data 1. *p<0.05; **p<0.01; ***p<0.001; *n.s.*, p>0.05.

**Table 1.** Intrinsic electrophysiological properties of Sst-INs, FS-INs, and PCs in visual cortex.

	Postnatal day	R_in_ (MΩ)	Threshold (mV)	Amplitude (mV)	Half-width (ms)	AHP (mV)
Sst-IN	P12-13 (n = 46)	430.2 ± 33.3	−51.7 ± 1.0	60.6 ± 0.9	2.46 ± 0.10	10.6 ± 0.4
	P14-15 (n = 27)	332.6 ± 26.6	−52.6 ± 1.3	61.0 ± 1.1	1.76 ± 0.16^***^	11.1 ± 0.5
	P17-20 (n = 30)	367.9 ± 27.9	−53.7 ± 1.4	60.0 ± 0.9	1.52 ± 0.07^***^	10.6 ± 0.5
FS-IN	P12-13 (n = 45)	201.8 ± 11.9	−44.0 ± 0.8	48.1 ± 0.8	1.45 ± 0.05	19.0 ± 0.3
	P14-15 (n = 18)	113.0 ± 8.7^***^	−45.0 ± 1.4	47.0 ± 1.1	1.03 ± 0.04^***^	18.4 ± 0.5
	P17-20 (n = 29)	126.9 ± 8.5^***^	−43.9 ± 1.0	49.4 ± 0.8	0.86 ± 0.04^***^	19.8 ± 0.4
PC	P12-13 (n = 32)	326.6 ± 20.6	−46.1 ± 1.3	63.8 ± 0.8	4.08 ± 0.13	11.4 ± 0.3
	P14-15 (n = 15)	214.0 ± 12.2^***^	−47.8 ± 1.8	65.6 ± 0.9	3.86 ± 0.30	11.5 ± 0.8
	P17-20 (n = 29)	212.4 ± 13.6^***^	−48.0 ± 1.2	68.8 ± 1.0^***^	3.45 ± 0.20^**^	12.1 ± 0.4

**p<0.01; ***p<0.001. P14-15 and P17-20 groups were compared with the P12-13 group.

**Figure 2.**
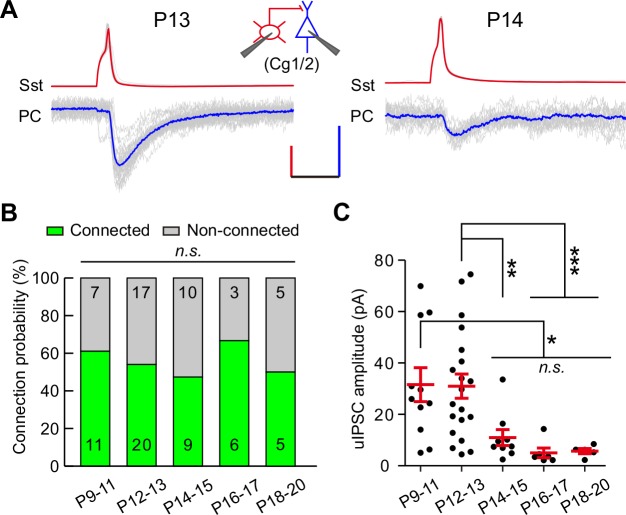
Development of synaptic transmission from Sst-INs onto PCs in the prefrontal Cg1/2 area. (**A**) Representative traces of synaptic transmission from an Sst-IN to a PC in layer 2/3 in the prefrontal Cg1/2 area at P13 and P14. Inset schema indicates paired patch recording of an Sst-IN and a PC. Scale bars: 50 mV (vertical, red), 20 pA (vertical, blue), and 20 ms (horizontal). (**B**) Histogram of the connection probability from P9 to P20. Data label indicates the number of pairs in each group. (**C**) Summary of the peak amplitude of uIPSCs from P9 to P20. Detailed statistical analysis, detailed data, and number of experiments are presented in Figure 2—source data 1. *p<0.05; **p<0.01; ***p<0.001; *n.s.*, p>0.05. 10.7554/eLife.32337.010Figure 2—source data 1.Detailed statistical analysis, detailed data, exact sample numbers, and *p* values in Figure 2.

To assess whether other cortical areas undergo similar developmental changes, we measured the connection probability and strength of uIPSCs from Sst-INs to PCs in layer 2/3 of the prefrontal cingulate cortex area 1/2 (Cg1/2) during P9–20 (Figure 2). The connection probability remained comparable from P9 to P20 (χ^2^ test, p=0.857; Figure 2B). Consistent with the result observed in the visual cortex, the peak amplitude of Sst-IN→PC uIPSCs at P14–15 was significantly smaller than that at P12–13 in Cg1/2 (P9–11, 31.6 ± 6.6 pA; P12–13, 31.0 ± 4.7 pA; P14–15, 11.0 ± 3.1 pA; P16–17, 5.0 ± 1.9 pA; P18–20, 5.7 ± 1.0 pA; one-way ANOVA, *F*_(4,46)_ *=* 5.51, p=6.0 × 10^−4^; Figure 2C). These results suggest that the weakening of Sst-IN→PC synaptic transmission during eye opening exists not only in the visual cortex but also in Cg1/2.

Together, these results demonstrate that coinciding with eye opening, Sst-IN→PC synaptic transmission dramatically weakens in cortical layer 2/3.

### FS-IN→PC synaptic transmission increases during eye opening

**Figure 3.**
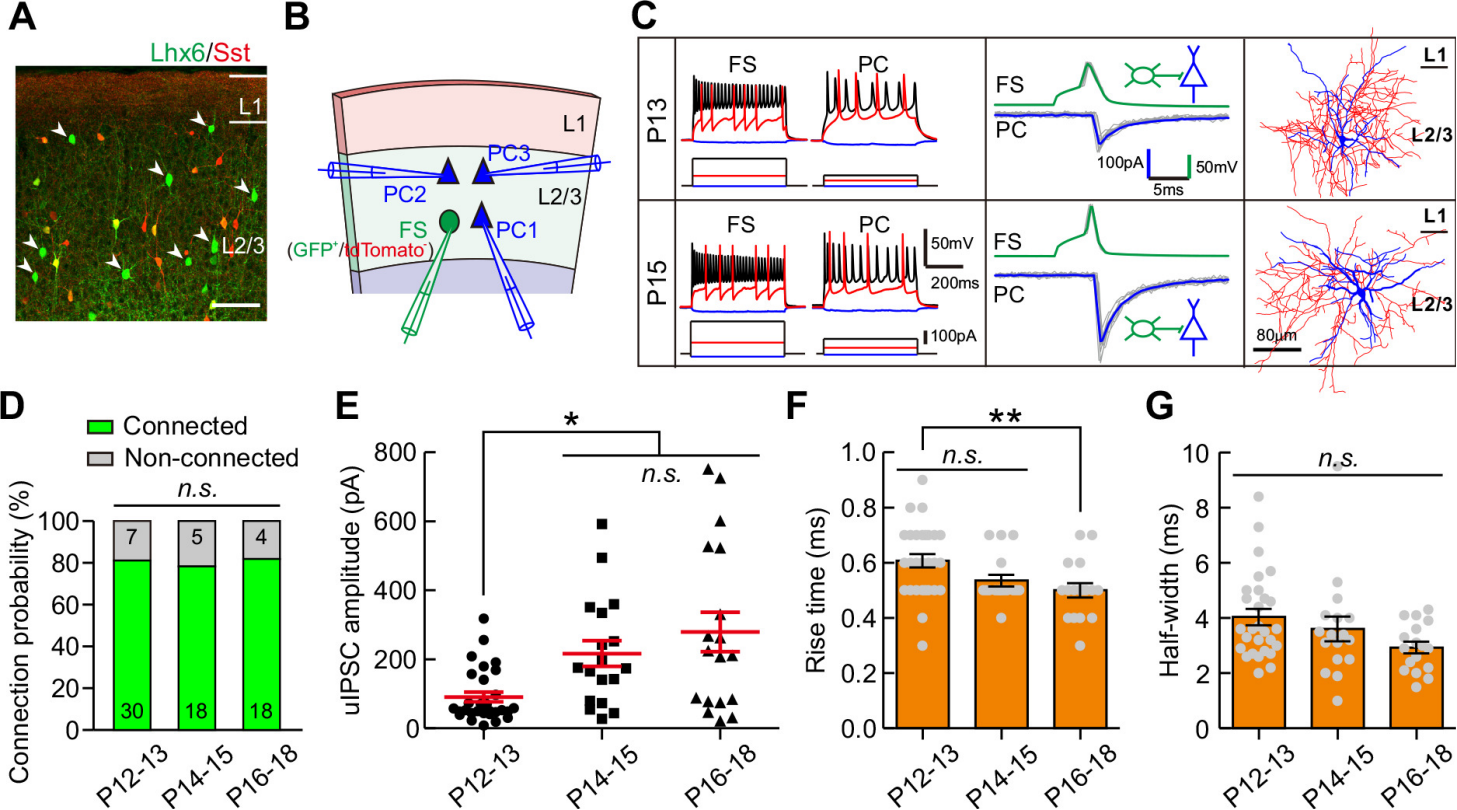
Strength of synaptic transmission from FS-INs onto PCs significantly increases in layer 2/3 of the visual cortex during eye opening. (**A**) Fluorescent image of a visual coronal section from *Sst-tdTomato::Lhx6-EGFP* line. TdTomato, Sst-INs; EGFP, Lhx6-EGFP cells. Arrowheads indicate EGFP^+^/tdTomato^-^ cells. Scale bar, 100 μm. (**B**) Schema of a quadruple whole-cell recording from an FS-IN (EGFP^+^/tdTomato^-^) and three PCs in layer 2/3. (**C**) Two examples of connection from an FS-IN to a PC at P13 and P15. *Left panels,* membrane potential responses of recorded FS-INs and PCs to current injections. *Middle panels*, synaptic transmission from FS-INs to PCs. *Right panels*, the reconstructed morphology of recorded FS-INs. (**D**) The connection probability from FS-INs to PCs did not change from P12 to P18. Data label indicates the number of pairs in each group. (**E**) The peak amplitude of FS-IN→PC uIPSCs at P14–15 and P16–18 was significantly larger than that at P12–13. (**F–G**) Quantification of the 10–90% rise time (**F**) and half-width (**G**) of uIPSCs at P12–18. Detailed statistical analysis, detailed data and number of experiments are presented in Figure 3—source data 1. *p<0.05; **p<0.01; *n.s.*, p>0.05. 10.7554/eLife.32337.014Figure 3—source data 1.Detailed statistical analysis, detailed data, exact sample numbers, and *p* values in Figure 3 and Figure 3—figure supplement 2.

We further examined whether the strength of synaptic transmission from fast-spiking PV interneurons (FS-INs) to PCs (FS-INs→PCs) could change during eye opening. We took advantage of *Lhx6-EGFP* transgenic mice, in which the majority of MGE-derived INs were labeled by EGFP. We bred this line onto *Sst-tdTomato* line (*Sst-tdTomato::Lhx6-EGFP* line) to distinguish Sst-INs (tdTomato^+^) from other types of INs (EGFP^+^/tdTomato^-^) (Figure 3A) (Tuncdemir et al., 2016). EGFP^+^/tdTomato^-^ FS-INs were further determined with the fast-spiking properties (Figure 3C, Figure 3—figure supplement 1 and Table 1). We found that 78.4% of recorded EGFP^+^/tdTomato^-^ cells were FS-INs at P12–18 (87 out of 111, Figure 3—figure supplement 1), and these FS-INs showed basket cell morphology (15 out of 15, Figure 3—figure supplement 1). To examine FS-IN→PC synaptic transmission, an EGFP^+^/tdTomato^-^ FS-IN and nearby tdTomato^-^/EGFP^-^ PCs were simultaneously recorded in layer 2/3 of the primary visual cortex (Figure 3B and C). The connection probability did not significantly change from P12 to P18 (χ^2^ test, p=0.949; Figure 3D). Interestingly, unlike Sst-IN→PC synaptic transmission, the strength of FS-IN→PC uIPSCs at P14–15 and P16–18 was significantly stronger than that at P12–13 (P12–13, 91 ± 14 pA; P14–15, 217 ± 37 pA; P16–18, 279 ± 57 pA; one-way ANOVA, *F*_(2,63)_ = 8.71, p=4.6 × 10^−4^; Figure 3E), suggesting that eye opening may increase the FS-IN→PC synaptic transmission. Moreover, the 10–90% rise time and half-width of uIPSCs did not exhibit obvious change between P12–13 and P14–15, while the rise time was significantly shorter at P16–18 than at P12–13 (Figure 3F and G). In Cg1/2 area, we also observed a significantly larger peak amplitude of FS-IN→PC uIPSCs at P14–15 and P17–20 than that at P12–13 (Figure 3—figure supplement 2), while the connection probability was comparable among the three groups (Figure 3—figure supplement 2). Overall, these results demonstrate that FS-IN→PC synaptic transmission increases during eye opening.

**Figure 3—figure supplement 1.**
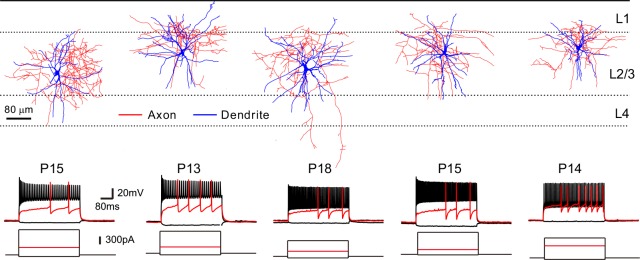
Morphologies of reconstructed FS-INs and corresponding evoked membrane responses. *Top panel*, the morphologic reconstructions of FS-INs. *Bottom panel*, the membrane responses of FS-INs to current injections.

**Figure 3—figure supplement 2.**
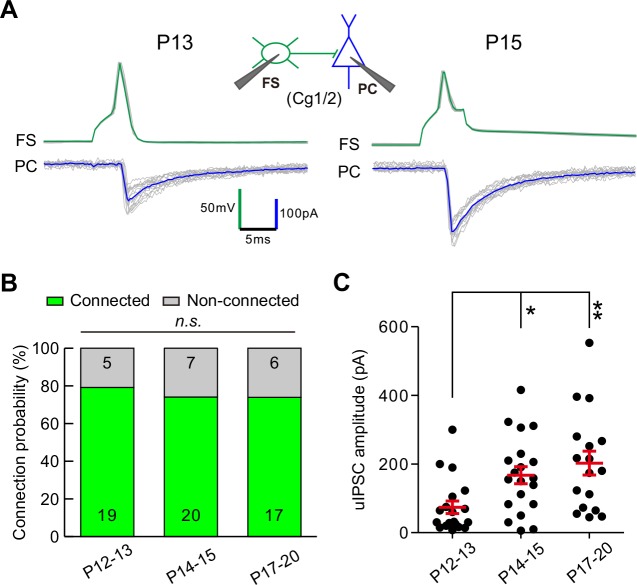
Synaptic transmission from FS-INs onto PCs increases in Cg1/2 area during eye opening. (**A**) Two examples of connection from an FS-IN onto a PC at P13 and P15 in Cg1/2 area. (**B**) Summary of the connection probability from FS-INs onto PCs. (**C**) The peak amplitude of FS-IN→PC uIPSCs significantly increased during eye opening. Detailed statistical analysis, detailed data, and exact sample numbers are presented in Figure 3—source data 1. *p<0.05; **p<0.01.

### Synaptic transmission from Sst-INs to other types of INs does not change during eye opening

**Figure 4.**
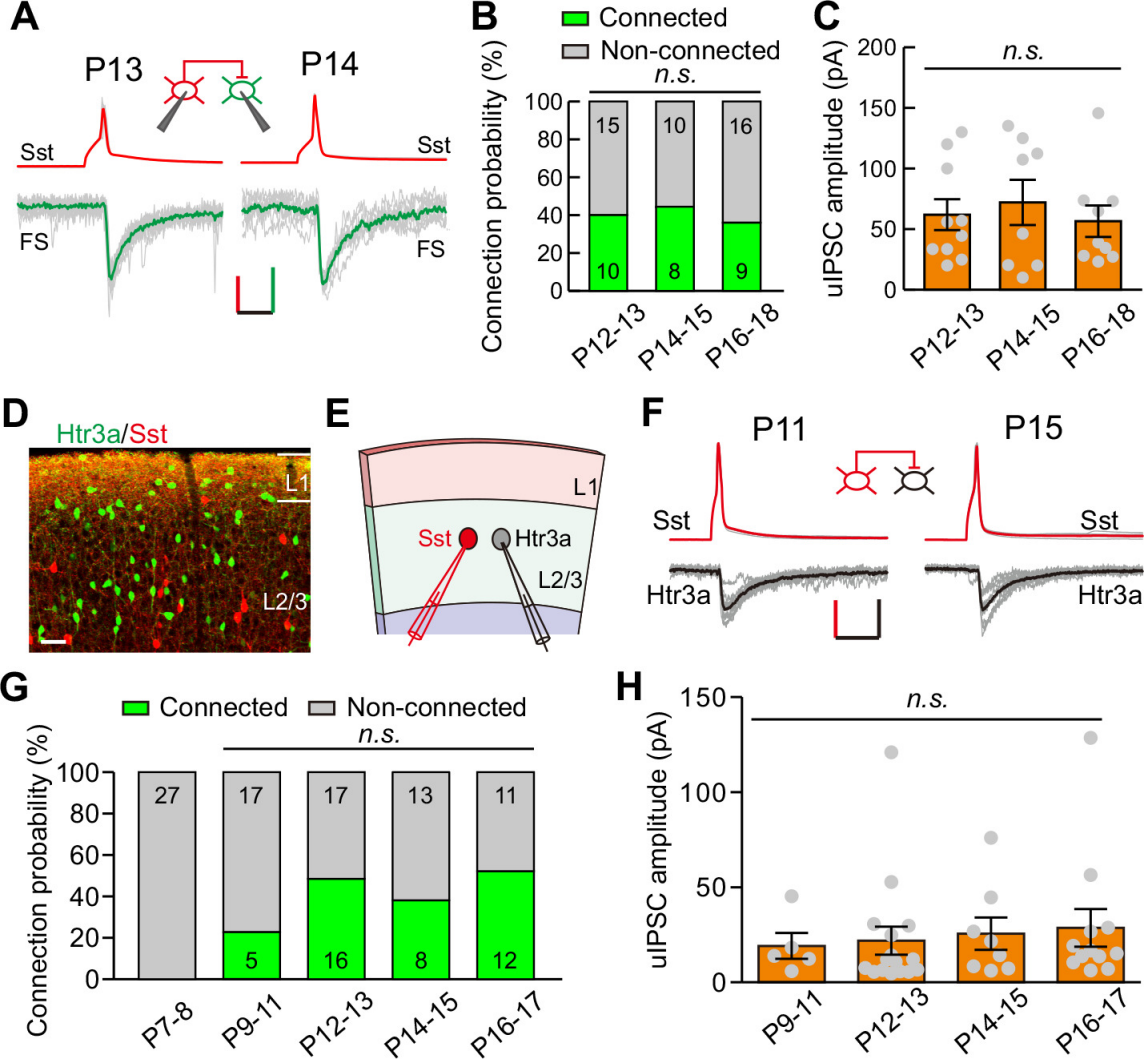
Development of synaptic transmission from Sst-INs to FS-INs and Htr3a-INs. (**A**) Representative evoked responses from an Sst-IN to an FS-IN in layer 2/3 at P13 and P14, respectively. *Inset panel*, schematic of a paired recording from an Sst-IN and an FS-IN. Scale bars: 50 mV (vertical, red), 25 pA (vertical, green), and 20 ms (horizontal). (**B**) Summary of connection probability from Sst-INs to FS-INs at different postnatal ages. A total of 68 pairs were recorded from 11 mice. Data label indicates the number of pairs in each group. (**C**) The peak amplitude of uIPSCs from Sst-INs to FS-INs was unchanged from P12 to P18. (**D**) Fluorescent image of a visual coronal section from *Sst-tdTomato::Htr3a-EGFP* line. TdTomato, Sst-INs; EGFP, Htr3a-INs. Scale bar, 50 μm. (**E**) Schematic of a paired recording from an Sst-IN (red) and an Htr3a-IN (gray) in layer 2/3. (**F**) Traces showing representative synaptic transmission from an Sst-IN and Htr3a-IN at P11 and P15. Scale bars: 40 mV (vertical, red), 20 pA (vertical, black), and 20 ms (horizontal). (**G**) Summary of connection probability from Sst-INs to Htr3a-INs at different postnatal ages. A total of 126 pairs were recorded from 17 mice. Data label indicates the number of pairs in each group. (**H**) The peak amplitude of uIPSCs from Sst-INs to Htr3a-INs did not significantly change from P9 to P17. Detailed statistical analysis, detailed data, and exact sample numbers are presented in Figure 4—source data 1. Error bars indicate mean ±SEM. *n.s.*, p>0.05. 10.7554/eLife.32337.017Figure 4—source data 1.Detailed statistical analysis, detailed data, exact sample numbers, and *p* values in Figure 4 and Figure 4—figure supplement 1.

Sst-INs not only frequently innervate pyramidal neurons, but also strongly inhibit other types of INs (Pfeffer et al., 2013). We first examined the development of GABAergic synaptic transmission from Sst-INs to FS-INs (Sst-INs→FS-INs) in *Sst-tdTomato::Lhx6-EGFP* line during eye opening. To study Sst-IN→FS-IN synaptic transmission, we simultaneously recorded a tdTomato^+^ Sst-IN and a nearby EGFP^+^/tdTomato^-^ FS-IN from layer 2/3 (Figure 4A). We compared the connection probability and strength of Sst-IN→FS-IN uIPSCs at P12–13, P14–15, and P16–18 (Figure 4B and C). Our data showed that the Sst-IN→FS-IN connection probability did not change from P12 to P18 (χ^2^ test, p=0.855; Figure 4B). Notably, the strength of Sst-IN→FS-IN uIPSCs did not exhibit any obvious change (P12–13, 62.0 ± 12.7 pA; P14–15, 72.1 ± 18.7 pA; P16–18, 56.6 ± 13.0 pA; one-way ANOVA, *F*_(2,24)_ = 0.268, p=0.767, Figure 4C). These results suggest that the strength of Sst-IN→FS-IN synaptic transmission does not change during eye opening.

**Figure 4—figure supplement 1.**
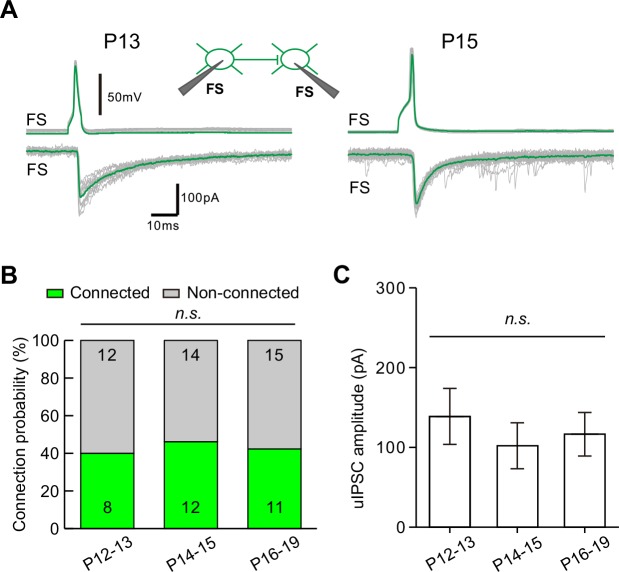
Synaptic transmission from FS-INs onto FS-INs does not change during eye opening. (**A**) Representative traces of FS-IN→FS-IN uIPSCs at P13 and P15. (**B**) The probability of chemical connections between FS-INs at different postnatal ages. (**C**) Summarized results of the peak amplitude of FS-IN→FS-IN uIPSCs. Detailed statistical analysis, detailed data, and exact sample numbers are presented in Figure 4—source data 1. *n.s.*, p>0.05.

We next explored the development of synaptic transmission from Sst-INs to Htr3a-positive interneurons (Htr3a-INs) in layer 2/3. Htr3a-INs are the predominant type of INs in the superficial visual layers, comprising ~50% of layer 2/3 INs (Lee et al., 2010). To simultaneously label Sst-INs and Htr3a-INs, we crossed heterozygous *Sst-tdTomato* mice with *Htr3a-EGFP* mice (*Sst-tdTomato::Htr3a-EGFP* line). Previous studies have demonstrated that 5HT3a receptors (encoded by *Htr3a*) in the neocortex are present exclusively in GABAergic INs (Lee et al., 2010). In *Sst-tdTomato::Htr3a-EGFP* mice, Sst-INs expressed tdTomato, while Htr3a-INs expressed EGFP (Figure 4D). Only ~1% of tdTomato^+^ Sst-INs expressed EGFP (1.1 ± 0.7%, six slices from three mice), suggesting that Sst-INs and Htr3a-INs are two different types of INs as previously reported (Chittajallu et al., 2013; Lee et al., 2010). To assess Sst-IN→Htr3a-IN synaptic transmission, we performed simultaneous recordings on pairs of tdTomato^+^ Sst-INs and EGFP^+^ Htr3a-INs in layer 2/3 of the primary visual cortex (Figure 4E and F). Sst-IN→Htr3a-IN connections emerged at P9–11, and the probability of connections did not change significantly from P9 to P17 (χ^2^ test, p=0.169; Figure 4G). Similar to Sst-IN→FS-IN uIPSCs, there was no obvious difference in the strength of Sst-IN→Htr3a-IN uIPSCs between P12–13 (before eye opening) and P14–15 (after eye opening) mice (P9–11, 19.1 ± 6.8 pA; P12–13, 21.9 ± 7.4 pA; P14–15, 25.6 ± 8.5 pA; P16–17, 28.6 ± 9.9 pA; one-way ANOVA, *F*_(3,37)_ = 0.183, p=0.907; Figure 4H). These results suggest that the strength of Sst-IN→Htr3a-IN synaptic transmission does not change during eye opening. Moreover, we found that the connection probability and strength of FS-IN→FS-IN uIPSCs exhibited no significant difference among P12–13, P14–15, and P16–19 groups (Figure 4—figure supplement 1).

**Figure 5.**
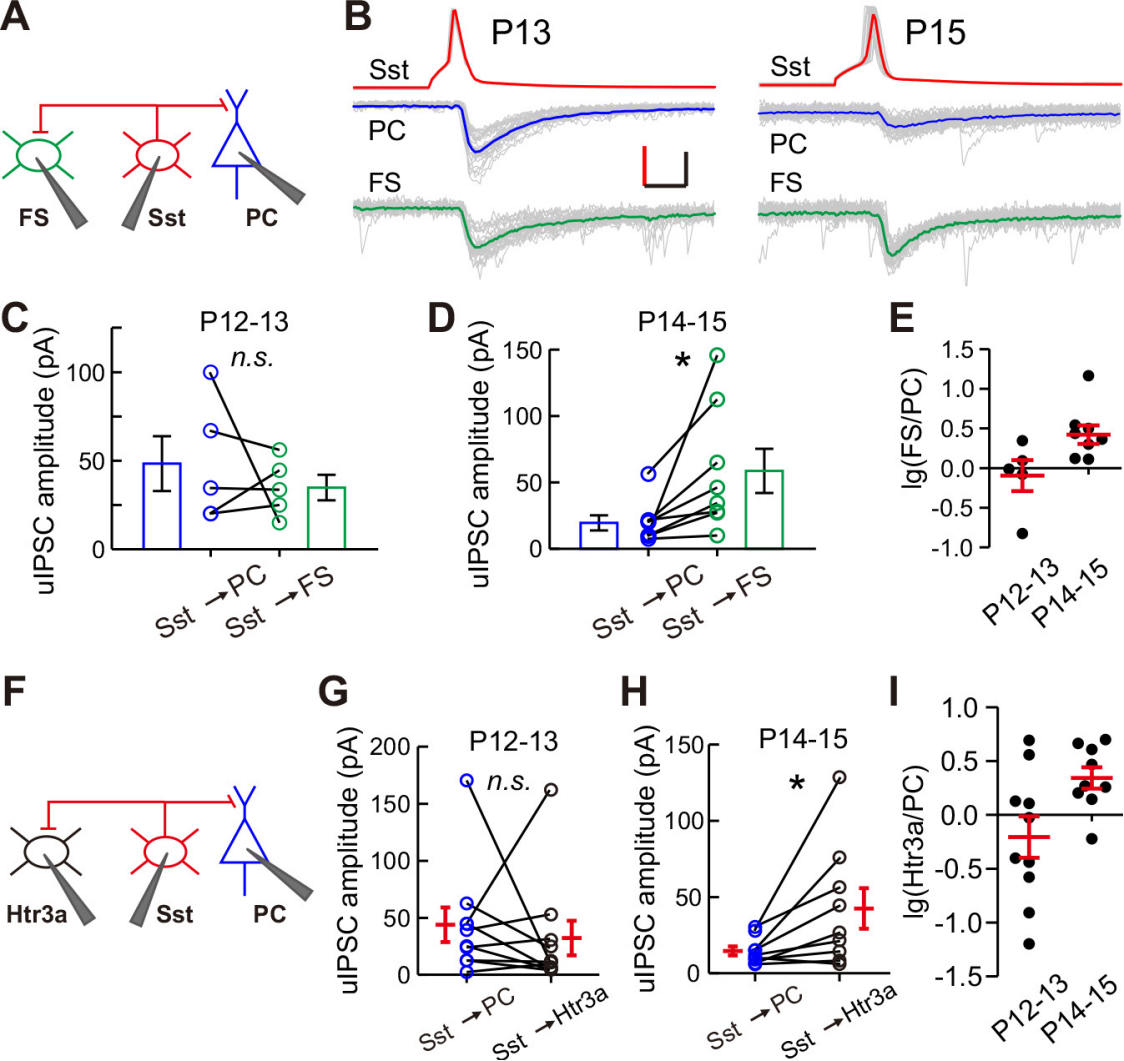
Differential inhibition by Sst-INs to various target cell types before and after eye opening. (**A**) Schematic of a triple recording from an Sst-IN, a PC, and an FS-IN. (**B**) Representative evoked responses from an Sst-IN simultaneously onto a PC and an FS-IN at P13 and P15. Scale bars: 50 mV (vertical, red), 50 pA (vertical, black), and 5 ms (horizontal). (**C**) uIPSC amplitude evoked by Sst-INs was not significantly different between PCs and FS-INs at P12–13. (**D**) uIPSC amplitude evoked by Sst-INs was significantly smaller in PCs than in FS-INs at P14–15. (**E**) The logarithm of the ratio between uIPSC amplitude in FS-INs and PCs at P12–13 and P14–15. (**F**) Schematic of a triple recording from an Sst-IN a PC and an Htr3a-IN. (**G**) uIPSC amplitude evoked by Sst-INs was not significantly different between PCs and Htr3a-INs at P12–13. (**H**) uIPSC amplitude evoked by Sst-INs was significantly smaller in PCs than in Htr3a-INs at P14–15. (**I**) The logarithm of the ratio between uIPSC amplitude in Htr3a-INs and PCs at P12–13 and P14–15. Detailed statistical analysis, detailed data, and exact sample numbers are presented in Figure 5—source data 1. Error bars indicate mean ±SEM. *p<0.05; *n.s.*, p>0.05. 10.7554/eLife.32337.019Figure 5—source data 1.Detailed statistical analysis, detailed data, exact sample numbers, and *p* values in Figure 5.

These results show that the strength of synaptic transmission from Sst-INs to PCs, but not from Sst-INs to other types of INs, selectively decreases in layer 2/3 of the cortex upon eye opening. To confirm the differential inhibition by Sst-INs onto various target cell types at different time points, we performed triple recordings to simultaneously record three cells: a Sst-IN and both a PC and a FS-IN in *Sst-tdTomato:: Lhx6-EGFP* mice (Figure 5A), or a Sst-IN and both a PC and a Htr3a-IN in *Sst-tdTomato::Htr3a-EGFP* mice (Figure 5F), at P12–13 and P14–15. The uIPSCs recorded simultaneously from two different postsynaptic cell types were directly compared. Sst-IN inhibition onto PCs and FS-INs exhibited no significant difference at P12–13 (paired *t*-test, p=0.510; Figure 5BC and E). However, at P14–15, the same Sst-INs produced much larger uIPSCs in FS-INs than in PCs (paired *t*-test, p=0.037; Figures 5B, D and E). Similarly, Sst-IN inhibition onto PCs versus that onto Htr3a-INs was not significantly different at P12–13 (paired *t*-test, p=0.601; Figure 5G and I). However, Sst-INs produced significantly larger uIPSCs in layer 2/3 Htr3a-INs than in PCs at P14–15 (paired *t*-test, p=0.037; Figure 5H and I). These results suggest that the inhibitory synapses formed by Sst-INs exhibit remarkable speciﬁcity in their connection strength with specific targets before and after eye opening.

### Eye opening modulates the Sst-IN→PC and FS-IN→PC synaptic transmission

Although the weakening of synaptic transmission from Sst-INs to PCs was observed at the time of eye opening, it could be induced by intrinsic developmental programs or other mechanisms with coincidental timing rather than by eye opening per se. To determine which factor is responsible for this regulation of synaptic transmission, we first deprived the visual inputs by dark rearing. We dark-reared *Sst-tdTomato* mice from P3 and recorded the synaptic transmission from Sst-INs to PCs in layer 2/3 of the primary visual cortex at P12–15 (Figure 6—figure supplement 1A). Under dark rearing condition, no significant changes were observed in connection probability (χ^2^ test, p=0.066; Figure 6—figure supplement 1C) nor unitary strength of Sst-IN→PC synaptic transmission between P12–13 and P14–15 (P12–13, 19.6 ± 4.1 pA, n = 13; P14–15, 21.7 ± 3.6 pA, n = 16; two-tailed unpaired *t*-test, p=0.702; Figure 6—figure supplement 1B and D). These results suggest that visual deprivation prevents the weakening of Sst-IN→PC synaptic transmission at the time of eye opening.

**Figure 6.**
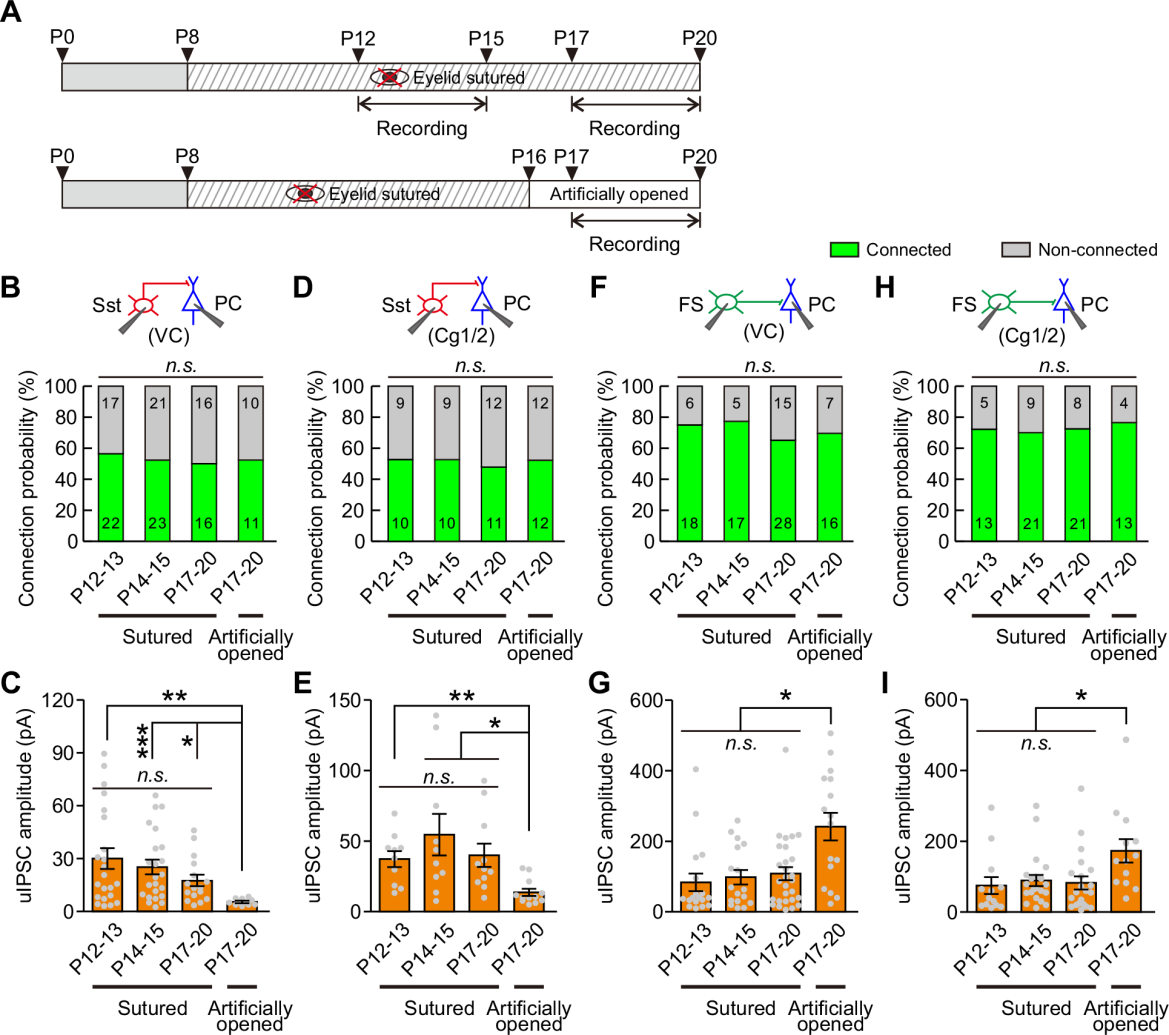
Eye opening modulates the strength of synaptic transmission from Sst-INs to PCs and from FS-INs to PCs. (**A**) Schematic schedule of eyelid suturing, eyelid reopening, and recording of synaptic connection. (**B**) Summary of Sst-IN→PC connection probability in visual cortex (VC). (**C**) Quantification of the peak amplitude of Sst-IN→PC uIPSCs in VC. (**D**) Summary of Sst-IN→PC connection probability in Cg1/2. (**E**) Quantification of the peak amplitude of Sst-IN→PC uIPSCs in Cg1/2. (**F**) Summary of FS-IN→PC connection probability in VC. (**G**) Quantification of the peak amplitude of FS-IN→PC uIPSCs in VC. (**H**) Connection probability from FS-INs to PCs in the Cg1/2 area from continuously sutured mice and reopened mice. (**I**) Summary of the peak amplitude of FS-IN→PC uIPSCs in Cg1/2. Data label indicates the number of pairs in each group. Detailed statistical analysis, detailed data, and exact sample numbers are presented in Figure 6—source data 1. Error bars indicate mean ±SEM. *p<0.05; **p<0.01; *n.s.*, p>0.05. 10.7554/eLife.32337.025Figure 6—source data 1.Detailed statistical analysis, detailed data, exact sample numbers, and *p* values in Figure 6 and Figure 6—figure supplements 1, 3 and 4.

Visual deprivation achieved by dark rearing not only blocks eye opening-induced visual inputs but also eliminates the natural diffuse dark/light stimulation presented through the eyelids before eye opening. Therefore, it is difficult to determine whether the elimination of the weakening of Sst-IN→PC synaptic transmission is induced by eye opening deprivation or by visual deprivation. To address this question, we performed binocular lid suture from P8 to block eye opening and manipulated the time of eye opening by artificially opening the lids in the binocular lid-sutured mice at P16 (two days after natural eye opening) (Figure 6A). The connection probability and strength of Sst-IN→PC and FS-IN→PC synaptic transmission were further compared in continuously sutured mice at P12–13, P14–15, P17–20 and those with eyelids artificially opened at P17–20 (i.e., different groups) (Figure 6 and Figure 6—figure supplement 2).

**Figure 6—figure supplement 1.**
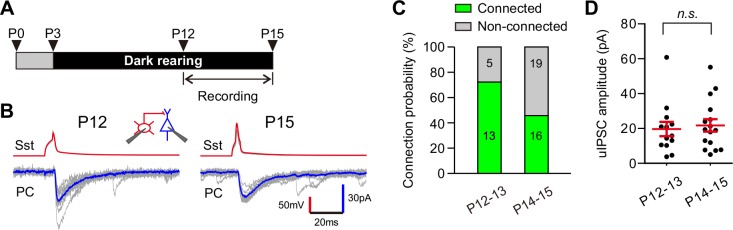
Dark rearing prevents the developmental decrease of synaptic transmission from Sst-INs to PCs. (**A**) Schematic schedule of dark rearing and electrophysiological recording. (**B**) Representative evoked responses from an Sst-IN to a PC at P12 and P15 in dark-reared mice. *Insert*, schematic of paired recording of an Sst-IN (red) and a PC (blue). (**C**) The occurrence of connection from Sst-INs to PCs in dark-reared mice was not significantly different between P12–13 and P14–15. (**D**) The peak amplitude of uIPSCs did not change significantly from P12–13 to P14–15 in dark-reared mice. Detailed statistical analysis, detailed data, and exact sample numbers are presented in Figure 6—source data 1. *n.s.*, p>0.05.

**Figure 6—figure supplement 2.**
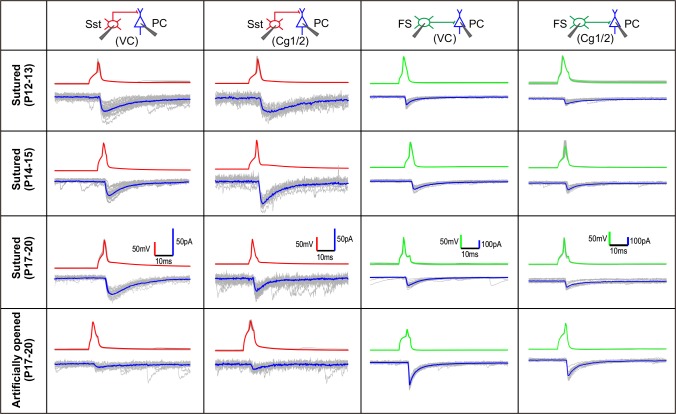
Representative traces of synaptic transmission (related to Figure 6). Top panels indicate the direction of synaptic transmission.

**Figure 6—figure supplement 3.**
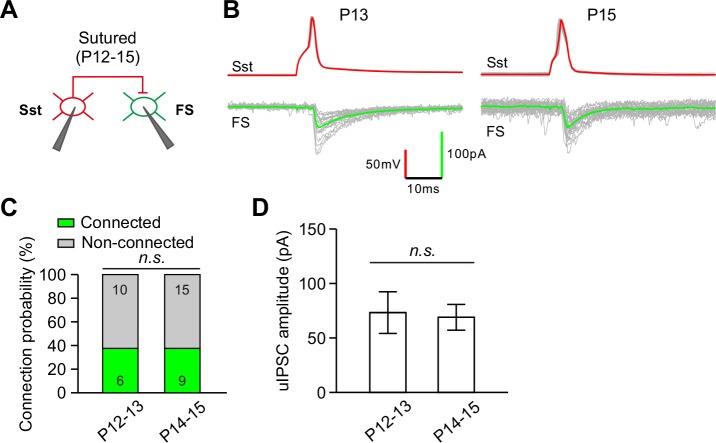
The strength of Sst-IN→FS-IN synaptic transmission does not change in continuously sutured mice during the time of eye opening. (**A**) Schema of a paired recording from an Sst-IN to an FS-IN in continuously sutured mice at P12–15. (**B**) Representative traces of synaptic transmission from an Sst-IN to an FS-IN at P13 and P15 in continuously sutured mice. (**C**) Quantification of the connection probability. (**D**) Quantification of the uIPSC peak amplitude. Detailed statistical analysis, detailed data, and exact sample numbers are presented in Figure 6—source data 1. *n.s.*, p>0.05.

**Figure 6—figure supplement 4.**
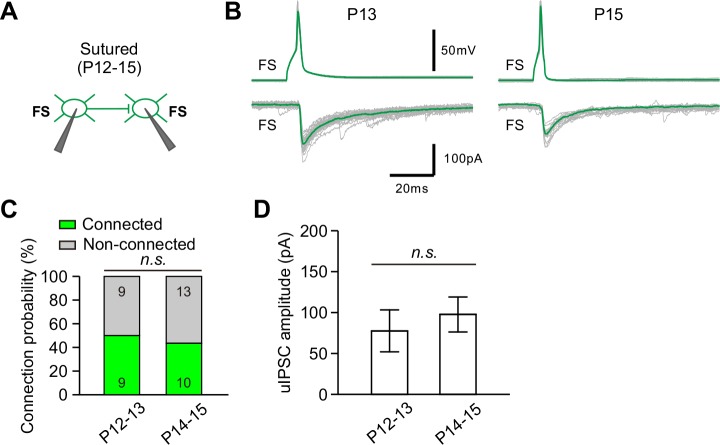
Suturing does not change the strength of FS-IN→FS-IN uIPSCs. (**A**) Schematic of paired recording of two FS-INs from sutured mice. (**B**) Representative traces of synaptic connections between two FS-INs at P13 and P15. (**C**) The connection probability between FS-INs at P12-13 and P14-15 from sutured mice. (**D**) Summarized results of averaged amplitude of FS-IN→FS-IN uIPSCs from sutured mice. Detailed statistical analysis, detailed data, and exact sample numbers are presented in Figure 6—source data 1. *n.s.*, p>0.05.

For Sst-IN→PC synaptic transmission within the visual cortex (VC), the connection probability was not significantly different among the different groups (χ^2^ test, p=0.958; Figure 6B). Consistent with dark rearing, there was no significant change in the peak amplitude of uIPSCs in continuously sutured mice between P12–13 and P14–15 (Figure 6C). Moreover, in continuously sutured mice, the strength of Sst-IN→PC uIPSCs at P17–20 was comparable to that at P12–13 or P14–15 (P12–13, 30.1 ± 5.9 pA; P14–15, 25.2 ± 4.1 pA; P17–20, 17.5 ± 10.6 pA; Figure 6C). These results suggest that eye opening deprivation prevents the weakening of Sst-IN→PC synaptic transmission. Interestingly, the strength of Sst-IN→PC uIPSCs at P17–20 in mice with eyelids artificially opened was significantly lower than that in continuously sutured mice at P12–13, P14–15, and P17–20 (one-way ANOVA, *F*_(3,68)_ = 4.231, p=0.008; Figure 6C). These results suggest that artificial eye opening can induce the weakening of Sst-IN→PC synaptic transmission and that this change associated with eye opening is unlikely due to an intrinsic developmental program. Notably, similar findings were observed in the prefrontal Cg1/2 area. In Cg1/2, the probability of Sst-IN→PC connections was not significantly changed among different groups (χ^2^ test, p=0.987; Figure 6D). The strength of Sst-IN→PC uIPSCs remained unchanged in continuously sutured mice at P12–13, P14–15, and P17–20 (Figure 6E). Furthermore, the strength of Sst-IN→PC uIPSCs at P17–20 in mice with eyelids artificially opened was significantly smaller than that in continuously sutured mice at P12–13, P14–15, and P17–20 (artificially eye-opened mice: P17–20, 13.6 ± 2.6 pA versus continuously sutured mice: P12–13, 37.3 ± 5.7 pA; P14–15, 54.6 ± 14.7 pA; and P17–20, 39.9 ± 8.3 pA; one-way ANOVA, *F*_(3,39)_ = 5.607, p=0.003; Figure 6E). In addition, both the connection probability and strength of Sst-IN→FS-IN synaptic transmission were comparable at P12–13 and P14–15 in continuously sutured mice (Figure 6—figure supplement 3).

For FS-IN→PC synaptic transmission in VC, similar to Sst-IN→PC synaptic transmission, there were no significant changes in the connection probability among the different groups (χ^2^ test, p=0.722; Figure 6F). Similarly, the strength of FS-IN→PC uIPSCs was similar in continuously sutured mice at P12–13, P14–15, and P17–20. In contrast, the strength in artificially eye-opened mice at P17–20 was significantly stronger than that in continuously sutured mice at P12–13, P14–15, and P17–20 (artificially eye-opened mice: P17–20, 241 ± 39 pA versus continuously sutured mice: P12–13, 84 ± 25 pA; P14–15, 98 ± 21 pA; and P17–20, 108 ± 19 pA; one-way ANOVA, *F*_(3,75)_ = 7.096, p=2.9 × 10^−4^; Figure 6G). In the Cg1/2 area, although the strength of FS-IN→PC uIPSCs in continuously sutured mice at P12–13, P14–15, and P17–20 was comparable, the strength in artificially eye-opened mice at P17–20 was also significantly stronger than that in continuously sutured mice at P12–13, P14–15, and P17–20 (Figure 6I). Furthermore, no significant changes in the connection probability were observed among the different groups (Figure 6H). In addition, both the connection probability and strength of FS-IN→FS-IN synaptic transmission were comparable at P12–13 and P14–15 in continuously sutured mice (Figure 6—figure supplement 4).

Together, these results strongly suggest that eye opening differentially modulates the strength of Sst-IN→PC and FS-IN→PC synaptic transmission.

### Eye opening alters postsynaptic quantal size

**Figure 7.**
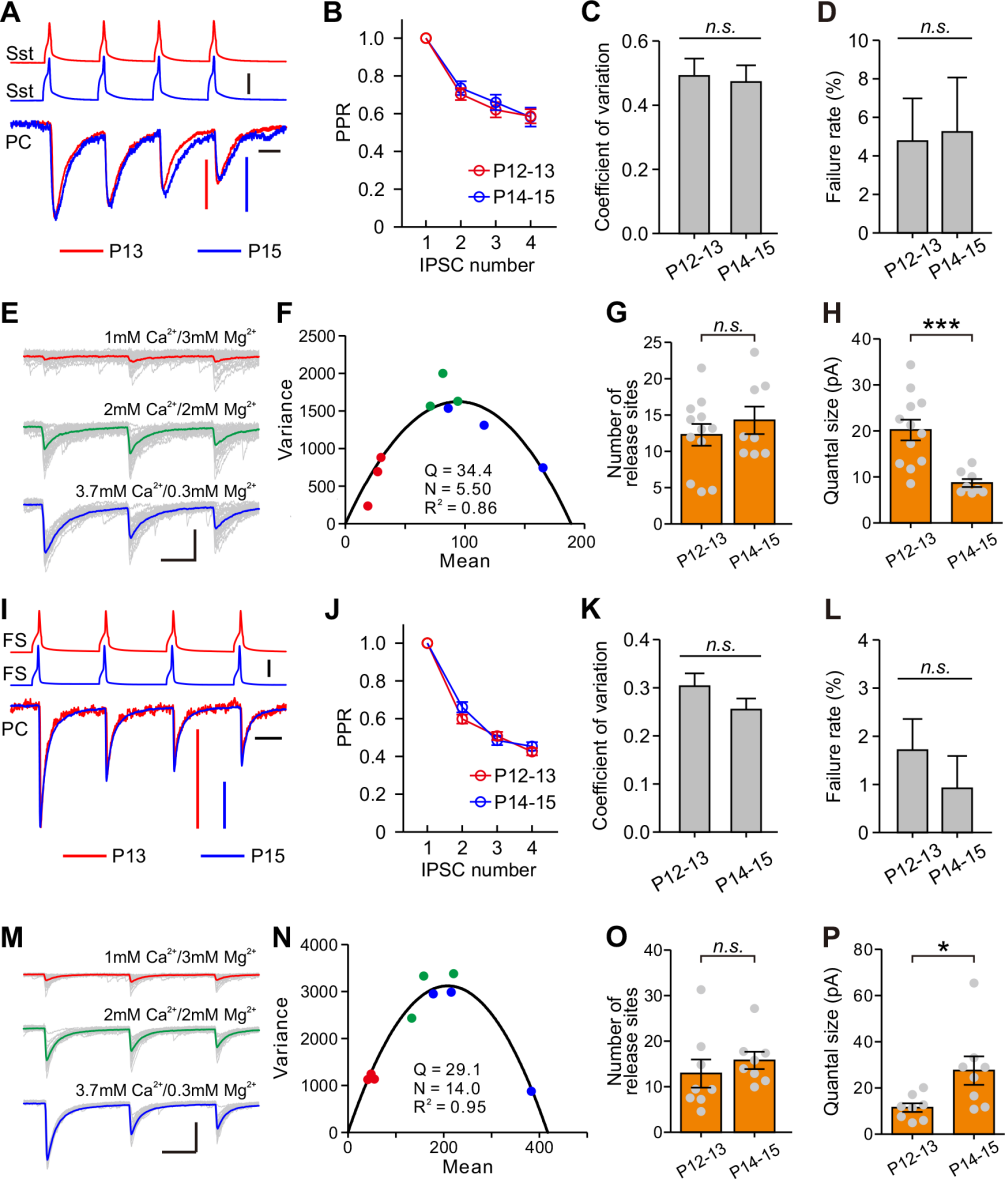
Postsynaptic mechanisms underlying the changes of synaptic transmission from Sst-INs to PCs and from FS-INs to PCs. (**A**) Amplitude-scaled overlay of paired-pulse ratio (PPR) responses in Sst-IN→PC connections at P13 and P15. Red, P13; blue, P15. Scale bars: 20 pA (vertical red), 10 pA (vertical, blue), 50 mV (vertical, black), and 20 ms (horizontal). Four presynaptic action potentials were evoked at 20 Hz. (**B**) The normalized peak amplitude of Sst-IN→PC uIPSCs showed short-term depression, and no significant difference in PPR was found between P12–13 (red) and P14–15 (blue) mice. (**C**) The coefficient of variation (C.V.) in Sst-IN→PC connections did not change from P12–13 to P14–15. (**D**) The failure rate in Sst-IN→PC connections did not change from P12–13 to P14–15. (**E**) Representative uIPSC responses from an Sst-IN to a PC evoked by a train of 3 presynaptic action potentials at 20 Hz under three different external Ca^2+^/Mg^2+^ concentrations. The postsynaptic cells were recorded with Cs-based and high Cl^-^ intracellular solution. Scale bars: 100 pA (vertical) and 20 ms (horizontal). (**F**) The parabola plot of the variance and mean of the peak amplitude of Sst-IN→PC uIPSCs in (**E**) under three different external Ca^2+^/Mg^2+^ concentrations. Red dots, 1 mM Ca^2+^/3 mM Mg^2+^; green dots, 2 mM Ca^2+^/2 mM Mg^2+^; blue dots, 3.7 mM Ca^2+^/0.3 mM Mg^2+^. (**G**) The number of release sites in Sst-IN→PC connections did not change from P12–13 to P14–15. (**H**) The quantal size in Sst-IN→PC connections significantly decreased from P12–13 to P14–15. (**I**) Amplitude-scaled overlay of paired-pulse ratio (PPR) responses in FS-IN→PC connections at P13 and P15. Red, P13; blue, P15. Scale bars: 100 pA (vertical red and blue), 50 mV (vertical, black), and 20 ms (horizontal). (**J**) PPR in FS-IN→PC connections was similar between P12–13 (red) and P14–15 (blue) mice. (**K**) The coefficient of variation (C.V.) in FS-IN→PC connections was unchanged from P12–13 to P14–15. (**L**) The failure rate in FS-IN→PC connections did not change from P12–13 to P14–15. (**M**) Representative uIPSC responses from an FS-IN to a PC evoked by a train of 3 presynaptic action potentials at 20 Hz in different external Ca^2+^/Mg^2+^ concentrations. Scale bars: 200 pA (vertical) and 20 ms (horizontal). (**N**) The parabola plot of the variance and mean of uIPSC amplitude in (**M**) at different external Ca^2+^/Mg2^+^ concentrations. Red dots, 1 mM Ca^2+^/3 mM Mg^2+^; green dots, 2 mM Ca^2+^/2 mM Mg^2+^; blue dots, 3.7 mM Ca^2+^/0.3 mM Mg^2+^. (**O**) The number of release sites in FS-IN→PC connections did not change from P12–13 to P14–15. (**P**) The quantal size in FS-IN→PC connections significantly increased from P12–13 to P14–15. Detailed statistical analysis, detailed data, and exact sample numbers are presented in Figure 7—source data 1. Error bars indicate mean ±SEM. *p<0.05; ***p<0.001; *n.s.*, p>0.05. 10.7554/eLife.32337.027Figure 7—source data 1.Detailed statistical analysis, detailed data, exact sample numbers, and *p* values in Figure 7.

We next examined presynaptic or postsynaptic mechanisms underlying the differential changes of Sst-IN→PC and FS-IN→PC synaptic transmission during eye opening. We assessed the presynaptic release probability by analysis of paired-pulse ratio (PPR), the coefficient of variation (C.V.), and failure rate (Miao et al., 2016; Pouzat and Hestrin, 1997). In Sst-IN→PC synaptic transmission, there were no significant differences in PPR (two-way ANOVA, *F*_(2,132)_ = 0.172, p=0.842; Figure 7A and Figure 7B), C.V. (P12–13, 0.494 ± 0.052, n = 20; P14–15, 0.472 ± 0.052, n = 23; two-tailed unpaired *t*-test, p=0.775; Figure 7C), or failure rate (P12–13, 4.8 ± 2.2%, n = 20; P14–15, 5.3 ± 2.8%, n = 23; two-tailed unpaired *t*-test, p=0.897; Figure 7D) before and after eye opening at P12–13 and at P14–15. These results suggest that eye opening does not affect the presynaptic release probability in Sst-IN→PC synaptic transmission. The number of the presynaptic release sites (N) and the postsynaptic quantal size (Q) were estimated by the variance-mean (V-M) analysis (Mitra et al., 2011; Scheuss and Neher, 2001). To estimate N and Q, we used the theoretically expected parabolic relationship between the variance and mean of synaptic responses under multiple-pulse stimulation and different external Ca^2+^/Mg^2+^ concentrations (1 mM Ca^2+^/3 mM Mg^2+^, 2 mM Ca^2+^/2 mM Mg^2+^, or 3.7 mM Ca^2+^/0.3 mM Mg^2+^) (Mitra et al., 2011). A train of three action potentials (20 Hz, 30–40 trials) was elicited in presynaptic Sst-IN, and postsynaptic responses were recorded in PCs (Figure 7E). We used a Cs-based intracellular solution containing a high concentration of Cl^-^ (60 mM) to record the postsynaptic currents. The relationship between mean and variance under different external Ca^2+^/Mg^2+^ concentrations was fit into a parabola plot (see Materials and methods, Figure 7F). We found that N in presynaptic terminals at P12–13 was similar to that at P14–15 (P12–13, 12.3 ± 1.5, n = 12; P14–15, 14.3 ± 1.9, n = 8; two-tailed unpaired *t*-test, p=0.419, Figure 7G). However, Q at P14–15 was significantly lower than that at P12–13 (P12–13, 20.2 ± 2.2 pA, n = 12; P14–15, 8.7 ± 0.9 pA, n = 8; Mann Whitney *U* test, p=2.9 × 10^−4^; Figure 7H), suggesting that postsynaptic mechanisms contribute to the developmental weakening of Sst-IN→PC synaptic strength.

Similarly, in FS-IN→PC synaptic transmission, there were no significant differences in PPR, C.V., or failure rate between P12–13 and P14–15 mice (Figures 7I, J, K and L). Moreover, the average values of N at P12–13 and P14–15 were not significantly different (P12–13, 12.9 ± 3.1, n = 8; P14–15, 15.8 ± 1.9, n = 8; two-tailed unpaired *t*-test, p=0.439, Figure 7O). Unlike Sst-IN→PC connections, Q of FS-IN→PC connections at P14–15 was significantly larger than that at P12–13 (P12–13, 11.5 ± 1.9 pA, n = 8; P14–15, 27.5 ± 6.2 pA, n = 8; two-tailed unpaired *t*-test, p=0.026, Figure 7P).

Together, these results suggest the postsynaptic quantal sizes in both of Sst-IN→PC and FS-IN→PC synaptic transmission are altered during eye opening.

## Discussion

The plasticity of GABAergic circuitry in the visual critical period in the developing visual cortex has been extensively studied by manipulations that disrupt normal visual experience after eye opening (Griffen and Maffei, 2014; Hensch, 2005; Lefort et al., 2013; Maffei et al., 2004). However, the changes and plasticity of GABAergic circuitry during eye opening are still far from fully understood (Gandhi et al., 2005; Kuhlman et al., 2011; Maffei et al., 2004). In this study, we show the following: (1) eye opening weakens the synaptic transmission from Sst-INs to PCs, but increases the synaptic transmission from FS-INs to PCs; (2) the inhibitory synaptic transmission from Sst-INs to other types of interneurons remains unaltered during eye opening; (3) eye opening-induced alteration of the inhibitory synaptic transmission onto PCs is mediated by changes in postsynaptic quantal size.

We studied the formation of inhibitory synapses onto layer 2/3 PCs and focused on Sst-INs and FS-INs. Although *Sst-IRES-Cre* line has been widely used to study Sst interneurons in cortical layer 2/3 both in vivo and in vitro, the neurons targeted by this line are heterogeneous (Hu et al., 2013; Jiang et al., 2015). Indeed, we observed that ~5.2% of Sst-tdTomato neurons in cortical layer 2/3 of visual cortex express PV and ~17.2% of Sst-tdTomato neurons show the fast-spiking properties. In spite of this heterogeneity, after removing these fasting-spiking tdTomato^+^ neurons, we observed that the vast majority of tdTomato^+^ cells in cortical layer 2/3 of the neocortex are Martinotti cells (95%, 19 out of 20). We exploited the *Sst-tdTomato::Lhx6-EGFP* line and identified FS-INs by EGFP^+^/tdTomato^-^ and fast-spiking properties in this study. Unlike PV-INs that innervate the cell body and proximal dendrites of the layer 2/3 PCs, Sst-INs innervate the distal regions of PCs, including the apical dendrites (Chen et al., 2015; Di Cristo et al., 2004). Notably, the synaptic strength measured in our study represents the strength at the soma of the recorded neuron rather than at the contact sites. These somatic recordings undoubtedly underestimate the distal dendritic tonic currents due to attenuation within the dendrites and limited spatial reach of somatic voltage clamp (Williams and Mitchell, 2008). Since significant differences were observed in neither the total length and complexity of PC dendrites (Figure 1—figure supplement 3) nor the rise time and half-width of uIPSCs between P12–13 and P14–15 mice (Figure 1F and G), the relative change in the specific Sst-IN→PC connection strength is unlikely due to space-clamp bias. Moreover, the connection probability and strength of Sst-IN→PC uIPSCs exhibit similar developmental properties when we used a cesium-based intracellular solution (improve space clamp) containing a high concentration of Cl^-^ (increase the driving force of uIPSCs) to record the postsynaptic currents.

We systematically examined the development of synaptic connections from Sst-INs onto PCs in layer 2/3 of the visual cortex. We observed that Sst-IN→PC connections emerge at P7–8, and the probability increases from P7–8 to P9–11. After P11, we found a dense and stable innervation of Sst-INs onto PCs, suggesting their crucial role in network activity. The increase in connection probability (from P7–8 to P9–11) was observed before the decline in peak amplitude of Sst-IN→PC uIPSCs (from P12–13 to P14–15), suggesting that the morphological growth and synapse function may be two independent processes. The average probability of Sst-IN→PC connections (~58% within 100 µm at P12–20) observed in this study agreed with that obtained via paired recordings in layer 2/3 of the somatosensory cortex (~63%) (Xu et al., 2013). However, the connection probability (~58%) is lower than those obtained through two-photon photostimulation in layer 2/3 of the frontal cortex (~70% within 200 µm) (Fino and Yuste, 2011) and through paired recordings in layer 2/3 of the visual cortex (~100% within 100 µm) (Pfeffer et al., 2013). Nonetheless, the connection probability (~58%) is higher than those reported with double or triple patch-clamp recordings in layer 2/3 (~20%) (Thomson and Lamy, 2007; Yoshimura and Callaway, 2005), layer 4 (~40% at P14–15 and ~20% at P20–22) (Miao et al., 2016), and layer 5 (~3%) (Otsuka and Kawaguchi, 2009) of the visual cortex. These discrepancies may be due to regional differences, layer specificity, or methodological differences.

In addition, we observed that the development of inhibitory synaptic transmission onto PCs displays several distinct features in layer 2/3 of mouse neocortex. First, the strength of Sst-IN→PC synaptic connections is rapidly reduced by ~65% from P12–13 to P14–15. Although the developmental synaptic transmission from Sst-INs onto PCs has not been quantified systematically, previous work reported that connection strength from low-threshold spiking interneurons (putative Sst-INs) to excitatory spinal neurons increases from P12–13 to P14–15 in layer 4 of the rat somatosensory cortex (Long et al., 2005). In contrast, a recent study found that both the connection probability and strength of Sst-IN→PC synaptic connections decrease substantially from P14–15 to P20–22 within layer 4 of the mouse visual cortex (Miao et al., 2016). The discrepancies may be due to layer differences (see below). Unlike Sst-IN→PC pairs, our data show that the strength of FS-IN→PC synaptic inputs in layer 2/3 of visual cortex rapidly increases by ~140% from P12–13 to P14–15. However, FS-IN→PC unitary conductance was reported to remain unaltered after P8 in layer 5/6 of mouse visual cortex (Pangratz-Fuehrer and Hestrin, 2011). Similarly, Yang *et al*. observed that the strength of FS-IN→PC uIPSCs is not changed after P9 in layer 5/6 of mouse prefrontal cortex (Yang et al., 2014). These studies imply that FS-IN→PC synaptic transmission does not change in the cortical layer 5/6 during eye opening. In addition, the rapid change of synaptic transmission from Sst-INs and FS-INs onto PCs coincides with the onset of eye opening. However, we cannot determine the exact temporal sequence of the two events (change of synaptic transmission and eye opening) due to the variability in the timing of eye opening (1–2 d) and synaptic responses. Nonetheless, with a controlled eye opening paradigm (Lu and Constantine-Paton, 2004; Yoshii et al., 2003), it will be interesting to further explore the sequence of events within the first 24 hr after eye opening. Lastly, the weakening of Sst-IN→PC synaptic transmission and the strengthening of FS-IN→PC synaptic transmission during eye opening exist not only in layer 2/3 of the visual cortex but also in layer 2/3 of the prefrontal Cg1/2 area. Indeed, eye opening has been shown to affect hippocampal development, and early eye opening accelerated the maturation of synaptic strength (Dumas, 2004). Nevertheless, it still remains unclear whether these changes are induced by light-mediated factors (vision in general) or vision-related factors (e.g. mobility directly or indirectly induced by the siblings or mother).

A major finding from our recordings in layer 2/3 of the visual cortex is that natural eye opening regulates synaptic transmission from Sst-INs and FS-INs onto PCs. This conclusion is based on three lines of experimental evidence. Firstly, visual deprivation (dark rearing) at an early postnatal period can prevent the weakening of Sst-IN→PC synaptic transmission. Secondly, eye opening deprivation (binocular lid suture) can efficiently prevent the changes in both of Sst-IN→PC and FS-IN→PC synaptic transmission. More importantly, we controlled the timing of eye opening by artificially opening the lids from the binocular lid-sutured mice two days after natural eye opening (P16) and compared synaptic responses between siblings with and without eye opening. Our data show that artificially opening the eyes decreases Sst-IN→PC synaptic transmission and increases FS-IN→PC synaptic transmission. These results strongly suggest that eye opening can specifically regulate the Sst-IN→PC and FS-IN→PC synaptic transmission in layer 2/3 of the visual cortex. Of note, a recent study found that although the synaptic strength of Sst-IN→PC connection significantly decreases from P14–15 to P20–22 within layer 4 of the mouse visual cortex, dark rearing does not affect the weakening of Sst-IN→PC synaptic transmission (Miao et al., 2016). Moreover, it has been reported that brief monocular visual deprivation during the time of eye opening selectively reduces the strength of synaptic transmission from PV-INs to PCs in layer 4 of the visual cortex (Maffei et al., 2004). These results suggest that visual deprivation-induced change in GABAergic circuits is layer- and cell type-specific. Indeed, accumulating evidence suggests that there are striking differences in morphology, intrinsic electrophysiological properties, and synaptic connectivity between layer 2/3 and layer 4 Sst-INs (Ma et al., 2006; Xu et al., 2013).

Interestingly, no significant changes were observed in the peak amplitude of uIPSCs from Sst-INs onto FS-INs and Htr3a-INs, and from FS-INs onto FS-INs. Moreover, eyelid suture does not change the peak amplitude of Sst-IN→FS-IN uIPSCs and FS-IN→FS-IN uIPSCs. These results suggest that within layer 2/3 of the visual cortex, the inhibitory synapses made by Sst-INs and FS-INs exhibit remarkable speciﬁcity in their strength with speciﬁc targets during eye opening. It should be noted that Htr3a-INs encompass a heterogeneous population of INs, including Vip-INs, Npy-INs, and other types of INs (Chittajallu et al., 2013; Lee et al., 2010; Pfeffer et al., 2013). It will be interesting to further investigate the development of synaptic transmission from Sst-INs to various subtypes of Htr3a-INs during eye opening.

Our data suggest that postsynaptic mechanisms may contribute to the developmental change of both Sst-IN→PC and FS-IN→PC synaptic strength. It is well known that GABA_A_ receptors (including different subunits) mediate the majority of inhibitory synaptic transmission in the mammalian cortex (Bowery and Smart, 2006). In addition, different subunits of GABA_A_ receptor are selectively inserted at specific GABAergic synapses (Ali and Thomson, 2008; Nusser et al., 1996). Moreover, previous studies indicated that the expression of GABA_A_ receptor subunits in neocortex changes significantly during early postnatal development (Bowery and Smart, 2006; Fritschy et al., 1994; Heinen et al., 2004). These findings suggest that the changes in subunit expression and/or composition of the GABA_A_ receptor may induce the differential developmental alternations of Sst-IN→PC and FS-IN→PC synaptic strength during eye opening.

**Figure 8.**
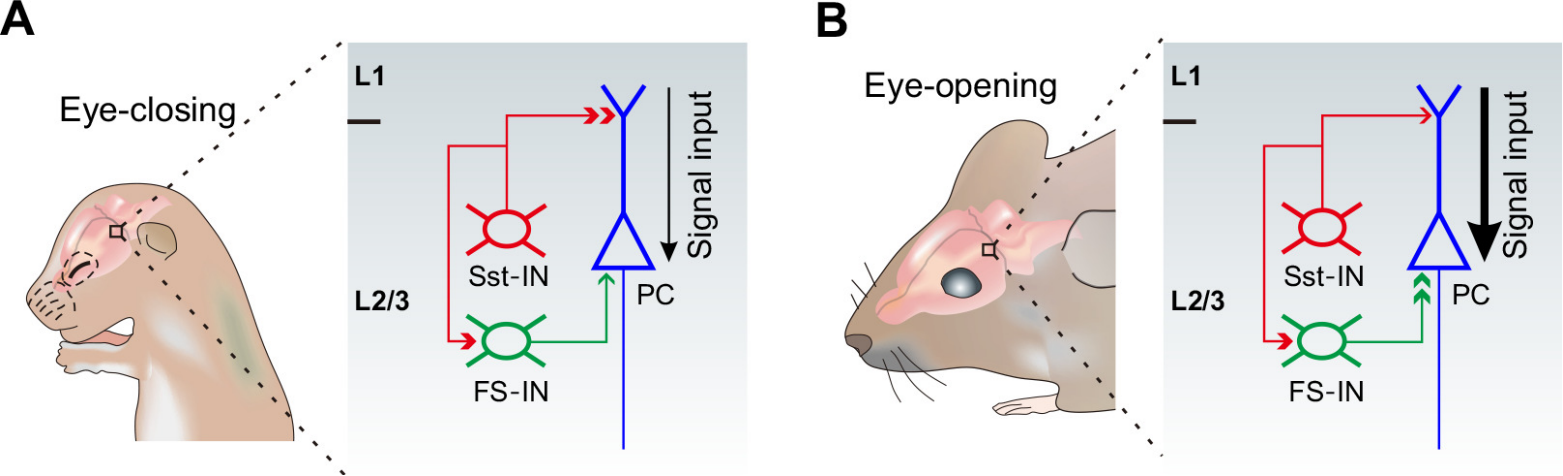
A model depicting how eye opening differentially regulates inhibitory synaptic transmission in developing layer 2/3 of the visual cortex. The development of inhibitory synaptic transmission before (**A**) and after eye opening (**B**). Sst-INs (red) primarily innervate distal dendrites of PCs (blue), while FS-INs (green) mainly target and inhibit somatic and perisomatic regions of PCs. Eye opening decreases the inhibition from Sst-INs to PCs. Decreased Sst-IN→PC inhibition after eye opening is predicted to enhance the effect of visual input (stronger signal input depicted by the thicker black arrow) onto excitatory neurons in the visual cortex by facilitating dendritic events. In contrast, synaptic inputs from FS-INs to PCs increase after eye opening. The increase of FS-IN→PC inhibition is speculated to involve in a homeostatic rebalancing of inhibition. The inhibition from Sst-INs to FS-INs remains unchanged during the time of eye opening.

The physiological roles for the differential alterations of inhibitory synaptic transmission onto PCs during eye opening remain unclear. Growing evidence suggests that Sst-INs that densely innervate nearby PC dendrites in mouse cortical layer 2/3 are responsible for controlling the efﬁcacy and plasticity of synaptic inputs (Chen et al., 2015; Chiu et al., 2013). Given that in early postnatal life, GABAergic transmission is excitatory to immature postsynaptic neurons (Ben-Ari, 2002; Owens et al., 1996), early emergence of Sst-IN→PC synaptic transmission may enhance the excitability of PCs, thereby promoting their maturation and synaptogenesis (Oh et al., 2016). However, around the time of eye opening, Sst-INs would inhibit PCs. Therefore, decreased Sst-IN→PC inhibition after eye opening could enhance the effect of visual input onto excitatory neurons in the visual cortex by facilitating dendritic events in distal regions (Figure 8). Contrary to Sst-INs, FS-INs control the spike output of PCs by inhibiting their perisomatic sites. Increased FS-IN→PC inhibition after eye opening is a homeostatic response to the reduction of Sst-IN inhibition and the resulting increase in the excitability of PCs (Bloodgood et al., 2013; Chen et al., 2015). We speculate that such enhanced visual input to the visual cortex gated by Sst-INs and the homeostatic rebalancing of inhibition regulated by FS-INs might be important for visual integration, an essential step in visual perception.

## Materials and methods

**Key resources table:** 

Reagent type (species) or resource	Designation	Source or reference	Identifiers	Additional information
Strain, strain background (*Mus musculus*)	*Sst-IRES-Cre*	PMID: 21943598	RRID: IMSR_JAX:013044	
Strain, strain background (*Mus musculus*)	*Htr3a-EGFP*	PMID: 14586460	RRID: MMRRC_000273-UNC	
Strain, strain background (*Mus musculus*)	*Lhx6-EGFP*	PMID: 14586460	RRID: MMRRC_000246-MU	
Strain, strain background (*Mus musculus*)	*Rosa-tdTomato*	PMID: 20023653	RRID: IMSR_JAX:007914	
Antibody	Anti-Red Fluorescent Protein	Rockland, USA	RRID: AB_2611063	1:500
Antibody	Anti-Green Fluorescent Protein	Aves, USA	RRID: AB_10000240	1:1000
Antibody	Anti-parvalbumin	Abcam, USA	RRID: AB_298032	1:500
Antibody	Donkey anti-mouse, Alexa Fluor 555 conjugated	Invitrogen, USA	RRID: AB_2536180	1:200
Antibody	Donkey anti-chicken, DyLight 488 conjugated	Jackson ImmunoResearch, USA	RRID: AB_2340376	1:200
Antibody	Donkey anti-rabbit, Alexa Fluor 488 conjugated	Life Technology, USA	RRID: AB_141708	1:200
Antibody	Cy5-Streptavidin	Jackson ImmunoResearch, USA	RRID: AB_2337245	1:500

### Animals

Four transgenic mouse lines, including *Sst-IRES-Cre* (RRID: IMSR_JAX:013044), *Htr3a-EGFP* (RRID: MMRRC_000273-UNC), *Lhx6-EGFP* (RRID: MMRRC_000246-MU), and tdTomato reporter *Ai14* (RRID: IMSR_JAX:007914) were used in this study. The *Sst-IRES-Cre* mice were crossed to *Ai14* mice to generate *Sst-tdTomato* alleles. The *Htr3a-EGFP* and *Lhx6-EGFP* mice were crossed to *Sst-tdTomato* mice to generate *Sst-tdTomato::Htr3a-EGFP* and *Sst-tdTomato::Lhx6-EGFP* alleles. All pups were reared under a normal 12 hr light/dark cycle unless otherwise stated. The day of parturition was defined as postnatal day 1 (P1). Pups were examined daily to monitor the postnatal day of eye opening. All experiments followed the guidelines for the care and use of laboratory animals at Fudan University.

### Dark rearing and eyelid suture

For dark rearing, pups were raised in dark cages after P3 until sacrificed for in vitro recordings. For eyelid suture, P8 mice were first carefully anesthetized with isoflurane and disinfected with ethanol. The binocular eyelids were sutured with small sterile ophthalmic needles. For artificial eye opening, sutured mice were anesthetized with isoflurane, and the eyelids were carefully opened at P16. After eyelid suture or artificial eye opening, the eyelids were covered with tetracycline ointment, and the pups were kept on warm blankets until fully recovered. If the eyelids of sutured mice were unexpectedly open before recording, the pups were discarded and not included in the recording experiment.

### Brain slice preparation

P5–20 mice were anesthetized with 1% isoflurane and 0.5–1.0 L/min oxygen. Brains from P5 to P8 mice were cut coronally at a thickness of 300 μm with a Compresstome VF-300 (Precisionary Instruments, USA) in a chilled solution containing (in mM) 120 choline chloride, 2.6 KCl, 26 NaHCO_3_, 1.25 NaH_2_PO_4_, 15 glucose, 1.3 ascorbic acid, 0.5 CaCl_2_, and 7 MgCl_2_ (pH 7.3–7.4, 300–305 mOsm). Brain slides were then incubated in artificial cerebrospinal fluid (ACSF) containing (in mM) 126 NaCl, 3 KCl, 1.25 KH_2_PO_4_, 1.3 MgSO_4_, 3.2 CaCl_2_, 26 NaHCO_3_, and 10 glucose (pH 7.3–7.4, 300–305 mOsm), bubbled with 95% O_2_/5% CO_2_. P9 to P20 brain slices were prepared by using a protective slicing and recovery method reported previously (Zhao et al., 2011). Briefly, anaesthetized mice were perfused intracardially with ice-cold oxygenated (95% O_2_, 5% CO_2_) NMDG-based cutting solution containing (in mM) 93 NMDG, 2.5 KCl, 1.2 NaH_2_PO_4_, 30 NaHCO_3_, 20 HEPES, 25 glucose, five sodium ascorbate, two thiourea, three sodium pyruvate, 10 MgSO_4_, 0.5 CaCl_2_, and 12 NAC (pH 7.3–7.4, 300–305 mOsm). Brains were carefully removed from the skull and cut coronally at a thickness of 300 μm with a Compresstome VF-300 in chilled oxygenated (95% O_2_, 5% CO_2_) NMDG-based cutting solution. Slices were initially recovered in NMDG-based cutting solution at 32°C for 10 mins. Slices were then incubated in oxygenated (95% O_2_, 5% CO_2_) HEPES-modified solution containing (in mM) 94 NaCl, 2.5 KCl, 1.2 NaH_2_PO_4_, 30 NaHCO_3_, 20 HEPES, 25 glucose, five sodium ascorbate, two thiourea, three sodium pyruvate, 2 MgSO_4_, 2 CaCl_2_, and 6 NAC (pH 7.3–7.4, 300–305 mOsm) at room temperature for 40 mins. Finally, slices were incubated in oxygenated (95% O_2_, 5% CO_2_) normal ACSF at room temperature for at least 1 hr before recording.

### Electrophysiological recording and analysis

Slices were transferred to a recording chamber, which was constantly perfused with fresh normal ACSF at 32–34°C, bubbled with 95% O_2_/5% CO_2_. Cells were visualized with water immersion objective (x20 and x60) and a BX51XI infrared-DIC microscope (Olympus, Japan) equipped with epifluorescence illumination. Glass recording electrodes (6–10 MΩ resistance) were filled with an intracellular solution consisting of (in mM) 93 K-gluconate, 16 KCl, 2 MgCl_2_, 0.2 EGTA, 10 HEPES, 2.5 MgATP, 0.5 Na_3_GTP, 10 Na-phosphocreatine, 0.4% neurobiotin (Invitrogen, USA), and 0.25% Alexa 568 or Alexa 488 (Invitrogen, USA) (adjusted to pH 7.25 and 295 mOsm). Cs-based intracellular solution contained (in mM) 65 cesium methanesulfonate, 60 cesium chloride, 10 HEPES, 4 MgATP, 0.3 Na_3_GTP, 0.5 EGTA, 10 Na-phosphocreatine, 0.4% neurobiotin, and 0.25% Alexa 488 (adjusted to pH 7.25 and 295 mOsm). Whole-cell recordings were obtained and analyzed by using two Axon Multiclamp 700B amplifiers, Digidata 1440A (Molecular Devices, USA), and pCLAMP10 software (Molecular Devices, USA). Signals were sampled at 5 kHz with a 2 kHz low-pass filter. Images were captured with an ORCA-R2 digital CCD camera (Hamamatsu, Japan). To test synaptic connections, we performed quadruple or triple whole-cell recordings. To test unitary transmission, we elicited a presynaptic action potential by injecting a brief suprathreshold current pulse (4–6 ms, 400–600 pA) intracellularly to the presynaptic cell. Postsynaptic cells were held at around −85 mV. Postsynaptic unitary responses were recorded 20–30 repeated sweeps at time intervals of 10 or 20 s. After that, a train of 4 or 10 current pulses (20 Hz) was injected into the presynaptic cell to assay the efficacy of synaptic transmission. After recording, slices were fixed in 4% paraformaldehyde (PFA) in PBS overnight at 4°C.

Recordings with R_s_ >30 MΩ were excluded from statistical analysis. R_s_ was compensated during recording. Electrophysiological data were analyzed off-line with Clampfit 10.6 (Molecular Devices). The rise time of uIPSCs was assayed from the 10% to 90% rising phase, and the half-width of uIPSCs was defined as the duration at the half amplitude. Input resistance (R_in_) was the slope of the linear regression of current-voltage response curve. Spike threshold was the membrane potential with a rise rate of 5 V/s. Spike amplitude was the voltage difference between the threshold and the peak of the action potential. Spike half-width was defined as the duration at half-maximum. Afterhyperpolarization (AHP) amplitude was the voltage difference between spike threshold and the maximal hyperpolarization following the spike. For the paired-pulse ratio calculation, the averaged peak amplitude of the first IPSC was defined as the basal level of synaptic strength. The C.V. was assayed from the amplitudes of each sweep. The failure rate was calculated as the percentage of sweeps without postsynaptic response. The fast-spiking physiological properties were identified as previously reported (Hu et al., 2013; Pangratz-Fuehrer and Hestrin, 2011). For immature neurons (P5–11), fast-spiking properties were characterized by subthreshold oscillations (Pangratz-Fuehrer and Hestrin, 2011).

The variance-mean analysis was performed as previously reported (Mitra et al., 2011; Scheuss and Neher, 2001). Recordings were first carried out in ACSF containing 2 mM Ca^2+^/2 mM Mg^2+^, and then the chamber solution was changed to ACSF containing 3.7 mM Ca^2+^/0.3 mM Mg^2+^ and 1 mM Ca^2+^/3 mM Mg^2+^. Trains of 3 action potentials at 20 Hz were elicited, and 30–40 repeated sweeps were recorded, with 10–20 s sweep-to-sweep interval. Recordings with stable baseline were used for analysis. The mean (*M*) and variance (*V*) of uIPSC amplitude were calculated for each pulse. The relationship between *M* and *V* was fitted to the parabola *V* = *QM − M*^2^/*N* (*Q*, quantal size; *N*, number of release sites). Quadratic regression was performed with GraphPad Prism five software (GraphPad Software). Only recordings with *R*^2^ >0.45 (*R*, regression index) were included for analysis.

### Immunohistochemistry and morphological reconstruction

Anesthetized *Sst-tdTomato::Htr3a-EGFP* and *Sst-tdTomato::Lhx6-EGFP* mice (P30) were transcardially perfused with cold phosphate buffered saline (PBS), followed by 4% paraformaldehyde (PFA) in PBS. Brains were carefully removed from the skull, post-fixed overnight at 4°C. The brains were rinsed in PBS and sectioned into 60 μm thick coronal slices with a VT1000S vibratome (Leica, Germany). After that, free-floating slices were incubated with primary antibodies in blocking solution (1% bovine serum albumin, 0.5% Triton X-100, and 0.05% sodium azide in PBS) for 48 hr at 4°C. Slices were then washed with PBST (0.1% Triton X-100 in PBS) five times (10 min each) and incubated in blocking solution containing secondary antibodies at 4°C for 24 hr. The primary antibodies included mouse anti-RFP (1:500, #200301379, Rockland, USA; RRID: AB_2611063), chicken anti-GFP (1:1000, #1020, Aves, USA; RRID: AB_10000240) and rabbit anti-parvalbumin (1:500, ab11427, Abcam, USA; RRID: AB_298032). The secondary antibodies were donkey anti-mouse (1:200, Alexa Fluor 555, A31570, Invitrogen, USA; RRID: AB_2536180), donkey anti-chicken (1:200, DyLight 488, #703-546-155, Jackson ImmunoResearch, USA; RRID: AB_2340376), and donkey anti-rabbit (1:200, Alexa Fluor 488, A21206, Life Technology, USA; RRID: AB_141708). For biocytin histochemistry, the fixed acute brain slices after electrophysiological recording were washed with PBS, incubated in blocking solution before incubation with Cy5-Streptavidin (1:500, #016-170-084, Jackson ImmunoResearch, USA; RRID: AB_2337245) for 48 hr. Finally, sections were washed in PBS five times (10 min each), mounted and cover-slipped. Confocal images were taken using an FV1000 confocal microscope (Olympus, Japan) with 20x objective and 1 μm z-step size. Neurons were then reconstructed with Neurolucida Software (MicroBrightField, USA).

### Quantification and statistical analysis

Data were analyzed with SPSS 22 software (IBM) and GraphPad Prism five software (GraphPad Software). Statistical significance between groups was tested by two-tailed one-sample *t-*test, two-tailed unpaired *t*-test, paired *t*-test, Mann-Whitney *U* test, Fisher’s exact test, χ^2^ test, one-way ANOVA and two-way ANOVA. All the detailed test methods, the number of experiments and *p* values are listed in the *source data*. All data are presented as mean ±SEM, and the difference was recognized as significant when p<0.05.

## Additional files

Transparent reporting form
