# Supplementary material for: Eye opening differentially modulates inhibitory synaptic transmission in the developing visual cortex
Source: eLife. 2017 Dec 11;6:e32337. doi: 10.7554/eLife.32337 (PMC5746341; doi:10.7554/eLife.32337)
Supplement: Peer review history

# Decision letter

**DOI:** 10.7554/eLife.32337.031

**Affiliations:** University of FreiburgUnited States

In the interests of transparency, eLife includes the editorial decision letter and accompanying author responses. A lightly edited version of the letter sent to the authors after peer review is shown, indicating the most substantive concerns; minor comments are not usually included.

[Editors’ note: a previous version of this study was rejected after peer review, but the authors submitted for reconsideration. The first decision letter after peer review is shown below.]

Thank you for submitting your work entitled "Eye opening selectively modulates inhibitory synaptic transmission in the developing visual cortex" for consideration by *eLife*. Your article has been reviewed by three peer reviewers, one of whom is a member of our Board of Reviewing Editors, and the evaluation has been overseen by a Senior Editor. The following individuals involved in review of your submission have agreed to reveal their identity: Simon J. B. Butt (Reviewer #2) and Alberto Bacci (Reviewer #3).

Our decision has been reached after consultation between the reviewers. Based on these discussions and the individual reviews below, we regret to inform you that your work will not be considered further for publication in *eLife*.

The study examines the postnatal maturation of connection probability and the kinetic characteristics of inhibitory signals at synapses of somatostatin-expressing interneurons (SSTI) targeting principal cells (PCs) as well as GABAergic interneurons (Htr3a-INs in layer 2/3 and INs in layer 1). The main observations are that connection probability of SSTIs increases onto both target PCs and INs as a function of postnatal development, in particular between P7/8 to P9/11. Moreover, the amplitude of unitary IPSCs selectively declines in target PCs between P12/13 and P14/15 at times when animals open their eyes (at ~P15). This observation is not selective for the visual cortex but could also be found in prefrontal brain areas. In contrast, peak amplitudes of unitary IPSCs at SSTI-Htr3a synapses and at SSTI-layer 1 IN synapses were unchanged. The authors propose that the decline in IPSC size depends on a reduction in the number of release sites and increase in quantal size. Moreover, pharmacological experiments indicate that additional postsynaptic mechanisms, particularly a decline in the expression of the GABA_A_ receptor subunit alpha5, play a role. The reviewers had major concern related to the technical standards of the recordings and the interpretation of the obtained data. Finally, they requested some additional experiments.

1) One major criticism was the high range of series resistances (Rs) of the recordings and the low Chloride concentration of the recording pipette solution resulting in a low driving force for chloride. Under these conditions it is very difficult to measure and quantify IPSC kinetics and finally to compare them at different times of postnatal development.

2. One reviewer was concerned about the lack of citations of new papers in the field relevant for the proposed study (see individual comments of the reviewers).

3. Concerns were raised in relation to the identity of the SST cells. Morphological reconstructions are missing as well as the proof that the applied SOm-Cre mouse line indeed results in the labelling of SSTs only, and no other types of neurons as recently reported. Physiological identification of the SSTs was also missing. Thus, a better SST-cell identification is required.

4. One reviewer was concerned with the interpretation of the data obtained from the cingulate cortex. These data allow the hypothesis that the change in the SST-IN to PC synapse is time locked across all cortical areas and NOT related to eye opening. The reviewer was asking whether other factors should also be considered. For completeness, it would have been good to study PV+ cells to examine whether or not the failure to reduce the amplitude of the SST-PC synapse could be compensating for reduced activity-dependent recruitment of this class of interneuron.

5. A major concern was formulated to data shown in Figure 5. They are not completely convincing. Indeed, a proper multiple-probability fluctuation analysis requires changes of extracellular Ca/Mg to estimate quantal synaptic parameters. The fit with a parabolic function of the data shown in Figure 5F is unconvincing, as all points cluster on the left side of the vertex. Moreover, how did the authors exclude receptor saturation and/or desensitization? This is especially problematic for synapses that are far from the recording sites (as is the case of distal dendritic synapses). Thus, a proper quantal analysis is needed.

Despite these criticisms, the reviewers judged the study as very interesting and therefore suggested that the authors may consider resubmitting their study once the major concerns have been addressed.

Reviewer #1:

The study examines the postnatal maturation of connection probability and the kinetic characteristics of inhibitory signals at synapses of somatostatin-expressing interneurons (SSTI) targeting principal cells (PCs) as well as GABAergic cells (Htr3a-INs in layer 2/3 and INs in layer 1). The authors focus these studies on the critical period at P14/5 during which eyes of mice are opened for the first time. The main observations are that connection probability of SSTIs increases onto both target PCs and INs as a function of postnatal development, in particular between P7/8 to P9/11. Moreover, the amplitude of unitary IPSCs selectively declines in target PCs between P12/13 and P14/15 at times when animals open their eyes (at ~P15). This observation is not selective for the visual cortex but could also be found in prefrontal brain areas. In contrast, peak amplitudes of unitary IPSCs at SSTI-Htr3a synapses and at SSTI-layer 1 INs, synapses were unchanged. The authors propose that the decline in IPSC size depends on a reduction in the number of release sites and increase in quantal size. Moreover, pharmacological experiments indicate that additional postsynaptic mechanisms particularly a decline in the expression of the GABA_A_ receptor subunit alpha5 play a role.

1) The mechanisms underlying a decline in IPSC size onto PCs is unclear. The authors propose a decline in the number of presynaptic release sites and at the same time an increase in the quantal content. A decline in the number of release sites should be reflected in an increased failure rate, independent on the quantal size. Moreover, the enhanced quantal content should be reflected in at least a trend to larger mIPSC amplitudes. Please explain.

2) A decline in the number of release sites is very interesting. However, additional confirmation such as electron microscopical investigations or immunohistochemical labelling of the location and the number of synaptic contact sites would be important to strengthen the conclusions. How does this compare to findings at P19/20 at almost full mature stages? Is there an additional change in synaptic function as proposed by Miao et al., 2016?

3) The same criticism applies to the alpha5 GABA_A_ receptor subunits. Additional confirmation of the pharmacological data would be very helpful in order to confirm the proposed conclusions. How selective is the applied agonist?

4) An important issue is the fact, that experiments have been performed at a series resistance (Rs) of up to 30 MOhm without Rs compensation. This broad range of Rs and its lacking compensation will have an enormous effect on the size of the measured IPSCs as well as the kinetic properties of IPSCs. The reviewer would therefore strongly recommend reducing the threshold of the Rs to ~15 MOHM and accordingly check whether the data allow the same conclusions.

5) It remains unclear why whole-cell recordings have been performed with a K-gluconate based intracellular solution containing ~20 mM Cl^-^. What was the experimentally determined reversal potential of IPSCs? It may have been a value of ~-65 mV (estimate of the reviewer). At a holding potential of -85 mV this will result in a driving force of only ~20 mV. This is a very small driving force for Cl^-^, for the main aim to measure differences in IPSC sizes.

6) Figure 4C and D show that the mean difference in the IPSC size between SST-PC connections at P12/13 and at P14/15 depends on few paired recordings in which the IPSC size at P12/13 was above 50 pA (n = 2 pairs). Please provide data on the distribution of IPSC sizes in both IPSC groups.

7) Increase in connectivity rises already at P9/11 prior to the decline in IPSC amplitude and reduced number of release sites at P14/15 suggesting that morphological growth and synapse function are here two independent processes. This should be emphasized and discussed in the manuscript.

8) The here shown data should be discussed in more detail with findings of Miao et al., 2016 who performed at least partly overlapping investigations in the visual cortex during postnatal development. In their study, reduced IPSC size correlated with reduced release sites and increased failure rate.

Reviewer #2:

The paper by Yu and colleagues is a worthy endeavour that uses multi-cell patch clamp electrophysiology to interrogate developing synaptic connections between somatostatin (SST)-positive interneurons and other neuronal subtypes, notably pyramidal cells, in the superficial layers of visual cortex. Such electrophysiology investigations are the 'gold standard' as they can provide high-resolution temporal assessment of synaptic kinetics.

1) The Introduction provides a reasonable summary of the field but the references cited could and should be updated to give the reader a more comprehensive and current view of the field. The following are just a few examples:

(i) In the first paragraph, it would apt to cite a number of recent papers on the subject of visual refinement post-eye opening, for example, Hoy & Niell, (2015) and work from the Mrsic-Flogel and Hoffer labs among many.

(ii) The authors state that our knowledge of how eye opening influences inhibitory neurotransmission is limited. However, it would be worth mentioning the work of Kuhlman et al., (2011) who investigated refinement of orientation bias in parvalbumin interneurons as an example of studies that have been performed to date.

(iii) The list of references cited here should be expanded: (i) include reference to Marques-Smith et al., (2016), who identified an early SST interneuron circuits in somatosensory cortex that forms a transient feed-forward GABAergic connections and (ii) include Oh et al., (2016) who demonstrated that GABA release from SST interneurons can promote synaptogenesis in mouse cortex.

Following these suggestions the authors might like to reconsider the statement and references cited in the Discussion section that "the changes and plasticity [of GABAergic circuits] during eye opening… has generally been neglected".

2) In the Results section: On a number of occasions the authors make vague reference to data that should be included to fully support their conclusions:

(i) I am uncomfortable with the declaration that the authors "explicitly" identified SST interneurons using the *Sst-ires-Cre* mouse in the absence of further data supporting this claim. There is evidence (Hu et al., 2013) that this GM mouse line labels and can be used to record fast spiking interneurons – typically associated with PV-expressing, basket cells. The authors state that cells were based on morphological and intrinsic electrophysiological data – it is important that this is shown in full.

(ii) Provide similar full data sets for the pyramidal cells.

3) Results section: The authors compare P12-P13 with P14-P15 (see for example Figure 1F,G). Please could they include the other time points recorded to give a more complete picture of the early IPSC kinetics? Comparison of these time points seems odd given the variability in eye-opening reported at P14 (Figure 1—figure supplement 2).

4) Results section: I am intrigued by the data from cingulate cortex. This is largely ignored in the rest of the manuscript – apart from a brief mention in the discussion – but would appear to suggest that the change in the SST-IN to PC synapse is time locked across all cortical areas and NOT related to eye opening? I appreciate that the subsequent data – for example the eye suturing experiments – support the eye opening-dependent hypothesis but perhaps there are other factors not considered? For completeness it would have been good to study PV+ cells to examine whether or not the failure to reduce the amplitude of the SST-PC synapse could be compensating for reduced activity-dependent recruitment of this class of interneuron. I appreciate that the authors suggest that these cells do not change after P9 (Discussion section) but these data are obtained from L5 visual cortex and prefrontal cortex. The argument put forward in the Discussion section for not recording PV+ cells is weak: The Hestrin lab and others have taken advantage of the G42 line to reliably identify putative-PV+ cells in neonatal cortex. A genetic strategy similar to that described on p.15 could have been employed. Indeed, it was likely that a large proportion of the cells labelled with GFP in the GAD-GFP line would have been putative PV+ cells. It is worth noting that Anastasiades et al., 2016 have shown that putative PV+, FS interneurons can be reliably differentiated from SST+, non-FS cells at early neonatal ages on the basis of a combined morphological and electrophysiological assessment.

5) Results section: The authors should acknowledge that the Htr3a line encompasses a much more diverse population than that studied by Pfeffer et al., (2013). It is important to acknowledge interneuron diversity and not assume that any of the GM lines used provide a homogeneous cohort in the absence of detailed characterization.

6) Results section: This data could be reasonably condensed or the rationale explained more clearly for why they recorded and separately report data from layer 1, then 2/3 then all the neuronal subtypes together.

7) Discussion section: This statement about eye-opening and development of other brain regions is not supported by the data presented in the current manuscript.

Reviewer #3:

In this manuscript, Guan and colleagues report an interesting observation, namely that SST-positive interneurons of mouse visual cortical layer 2/3 scale down their inhibitory synaptic responses, following eye opening and selectively on pyramidal neurons. The authors go on and show that this is not due to the actual intrinsic development of cortical circuits, but, rather, it depends on visual sensory activity. They conclude that this effect depends on a combination of pre- and post-synaptic mechanisms at GABAergic synapses between SST interneurons and layer 2/3 pyramidal neurons.

The manuscript is very well written, and the experiments are illustrated with a clear logic. The finding seems robust and quite interesting. The paper has, however, some limitations.

1) Use of *Sst-IRES-Cre* mouse: whereas this mouse line has been widely used to study SST interneurons in cortical layer 2/3 both in vivo and in vitro, cortical SST cells are heterogeneous (Scheyltjens and Arckens, 2016; Hu et al., 2013; Rossier et al., 2015). The authors claim that they restricted their analysis on Martinotti cells, but the evidence that they provide is anecdotal. Indeed, Figure 1—figure supplement 1 shows only one reconstructed neuron: It would be useful to know if that interneuron was representative of the entire sampled population. The authors should include a quantitative morphological analysis of axonal projections on some recorded neurons to corroborate their claim that they are recording Martinotti cells. Likewise, there is no quantification of the firing properties, but only two representative traces are shown (Figure 1—figure supplement 1B). If the authors want to make the point that they consistently record from Martinotti cells, they should provide more solid evidence.

2) The reduction of SST-P GABAergic synapses induced by eye opening seems to be target specific. This is indeed interesting, and the causal link between eye opening and synaptic weakening, is the core of the article. Importantly, however, potential non-specific effects induced by their manipulations should be ruled out. Target specificity of dark rearing and artificial eye opening at P17 is missing: it would be nice to see that dark rearing and/or eye surgery does not affect the other connections (SST-IN => L1-IN or SST-IN => L2/3 Htr3a-IN) as they showed for the developmental characterization.

3) It is not entirely clear to me why the authors chose layer 1 interneurons and 5-HT3a positive cells as other postsynaptic targets. For example, PV and VIP cells are also known to be connected by SST interneurons (Ma et al., 2012; Pfeffer et al., 2013).

4) The data shown in Figure 5 are not completely convincing. Indeed, a proper multiple-probability fluctuation analysis requires changes of extracellular Ca/Mg to estimate quantal synaptic parameters. The fit with a parabolic function of the data shown in Figure 5F is unconvincing, as all points cluster on the left side of the vertex. Moreover, how did the authors exclude receptor saturation and/or desensitization? This is especially problematic for synapses that are far from the recording sites (as is the case of distal dendritic synapses). Moreover, the authors recorded distant synapses using a K-based intracellular solution with relatively low intracellular chloride (~20 mM).

5) Effect of the alpha5-IA on synapses between SST-INs and P neurons: Interestingly, there is only a 10% effect of the drug after eye opening. This result does not fit with the data shown by Ali and Thomson, (2008), who recorded from rats after eye opening (P18-22). How do the authors explain these apparent contradicting results?

6) The results on cingulate cortex are somehow confusing, as in principle, this cortex does not receive direct visual information, and the authors' interpretation of their results is indeed a visual-mediated effect. Perhaps, the results on cingulate cortex should be either better discussed or removed.

[Editors’ note: what now follows is the decision letter after the authors submitted for further consideration.]

Thank you for submitting your article "Eye opening differentially modulates inhibitory synaptic transmission in the developing visual cortex" for consideration by *eLife*. Your article has been reviewed by three peer reviewers, one of whom is a member of our Board of Reviewing Editors, and the evaluation has been overseen by Eve Marder as the Senior Editor. The following individuals involved in review of your submission have agreed to reveal their identity: Alberto Bacci (Reviewer #2) and Simon J. B. Butt (Reviewer #3).

The reviewers have discussed the reviews with one another and the Reviewing Editor has drafted this decision to help you prepare a revised submission.

Summary:

By using paired or triple simultaneous recordings from somatostatin expressing presynaptic interneurons (SSIs) and target principal cells (PCs) or target fast-spiking interneurons or Htr3a-expressing interneuron in acute slice preparations of the visual and cingulate cortex the authors show that synaptic inhibitory SSI output signaling declines after eye opening onto PCs but not onto target interneurons. Moreover, fast-spiking interneuron-mediated signaling increases after eye opening onto PCs. This is caused by postsynaptic mechanisms and expressed as decline vs increase in the quantal content but not by presynaptic mechanisms such as release probability or number of release sites. Although experimentally well done, it remains unclear what factor induces the postsynaptic change in quantal size.

Essential revisions:

1) The new data with PV cells are important and add significant impact to the manuscript. The authors show that the developmental change of inhibitory strength from PV cells goes in the opposite direction than that of SST interneurons. The target-specific effects of SST-cell synapses are interesting and this raises the question of whether also PV-PV connections are not affected as PV-PC synapses. Therefore, please perform additional PV-PV paired recordings.

2) It remains unclear what factor induces the change in synaptic inhibitory strength. Since it is a postsynaptic effect, direct signaling mechanisms induced by the presynaptic interneuron are not likely to be involved (failure rate, release probability are unchanged). Since the main observation was obtained in both the visual and cingulate cortex, it remains unclear whether the here described main phenomenon has indeed something to do with light-mediated factors (vision in general) or vision-related factors such as mobility directly or indirectly induced by the siblings or mother. Therefore, please discuss more the effect of eye opening in cortical brain regions despite the visual cortex.

3) Improve the spelling.

4) Formulate a hypothesis what factor may cause the postsynaptic mechanisms supporting increase in the quantal content.

# Reply: Author response

**DOI:** 10.7554/eLife.32337.032

[Editors’ note: the author responses to the first round of peer review follow.]

Reviewer #1:

The study examines the postnatal maturation of connection probability and the kinetic characteristics of inhibitory signals at synapses of somatostatin-expressing interneurons (SSTI) targeting principal cells (PCs) as well as GABAergic cells (Htr3a-INs in layer 2/3 and INs in layer 1). The authors focus these studies on the critical period at P14/5 during which eyes of mice are opened for the first time. The main observations are that connection probability of SSTIs increases onto both target PCs and INs as a function of postnatal development, in particular between P7/8 to P9/11. Moreover, the amplitude of unitary IPSCs selectively declines in target PCs between P12/13 and P14/15 at times when animals open their eyes (at ~P15). This observation is not selective for the visual cortex but could also be found in prefrontal brain areas. In contrast, peak amplitudes of unitary IPSCs at SSTI-Htr3a synapses and at SSTI-layer 1 INs, synapses were unchanged. The authors propose that the decline in IPSC size depends on a reduction in the number of release sites and increase in quantal size. Moreover, pharmacological experiments indicate that additional postsynaptic mechanisms particularly a decline in the expression of the GABA_A_ receptor subunit alpha5 play a role.

1) The mechanisms underlying a decline in IPSC size onto PCs is unclear. The authors propose a decline in the number of presynaptic release sites and at the same time an increase in the quantal content. A decline in the number of release sites should be reflected in an increased failure rate, independent on the quantal size. Moreover, the enhanced quantal content should be reflected in at least a trend to larger mIPSC amplitudes. Please explain.

We thank the reviewer for raising this issue. We agree with reviewer that decline in the number of release sites should be reflected in an increased failure rate (the failure rate in this study is unchanged). We think the conflict should be largely due to the inaccurate quantal analysis/methods using in this study. In the revised manuscript, we used the theoretically expected parabolic relationship between the variance and mean of synaptic responses under different external Ca^2+^/Mg^2+^ concentrations to obtain the estimates of the presynaptic release sites (N) and the postsynaptic quantal size (Q). Moreover, we used a Cs-based intracellular solution (improve space clamp) containing high concentration of Cl^-^ (increase the driving force of uIPSCs) to record the postsynaptic currents. We found that the average values of N at P12–13 and P14–15 were not significantly different in both SST=>PC and FS=>PC synaptic transmission (Figure 7G and 7O). However, Q of SST=>PC synaptic transmission at P14–15 was significantly lower than that at P12–13 (Figure 7H); whereas we found a significant increase in Q at P12–13 compared with the mice at P14–15 in FS=>PC connections (Figure 7P).

In addition, the quantal content of SST=>PC connections in this study exhibited a decrease rather than an increase.

2) A decline in the number of release sites is very interesting. However, additional confirmation such as electron microscopical investigations or immunohistochemical labelling of the location and the number of synaptic contact sites would be important to strengthen the conclusions.

As mentioned above, we revised the quantal analysis/methods. We found that the average values of the presynaptic release sites at P12–13 and P14–15 were not significantly different in both SST=>PC and FS=>PC synaptic transmission (Figure 7G and 7O).

How does this compare to findings at P19/20 at almost full mature stages? Is there an additional change in synaptic function as proposed by Miao et al., 2016?

Although we conducted similar quantal analysis as Miao et al., 2016, there are obvious distinctions between two studies. First, Miao et al. analyzed the number of release sites in PC=>SST-IN excitatory synapses, whereas we studied SST-IN=>PC inhibitory synapses. Second, Miao et al. studied the synaptic transmission within layer 4 of the visual cortex, whereas we examined the synaptic transmission in layer 2/3 of the visual cortex.

3) The same criticism applies to the alpha5 GABA_A_ receptor subunits. Additional confirmation of the pharmacological data would be very helpful in order to confirm the proposed conclusions. How selective is the applied agonist?

We thank the reviewer for this valuable point. Given that the precise mechanism underlying the changes of SST-IN=>PC and FS-IN=>PC synaptic transmission during eye opening still remains largely elusive, we omitted α5-GABA_A_Rs-related data in the revised manuscript. Instead, we have studied the development of synaptic transmission from fast-spiking PV interneurons (FS-INs) to pyramidal cells (PCs) in layer 2/3 of the visual cortex (Figure 3). Interestingly, unlike synaptic transmission from SST interneurons (SST-INs) to PCs, the strength of FS-IN=>PC synaptic inputs in layer 2/3 of visual cortex was rapidly increased by ~140% from P12–13 (before eye opening) to P14–15 (after eye opening) (Figure 3E). More importantly, we further demonstrated that the dramatic increase can be prevented by binocular lid suture and reproduced by artificial opening of sutured lids (Figure 6G).

4) An important issue is the fact, that experiments have been performed at a series resistance (Rs) of up to 30 MOhm without Rs compensation. This broad range of Rs and its lacking compensation will have an enormous effect on the size of the measured IPSCs as well as the kinetic properties of IPSCs. The reviewer would therefore strongly recommend reducing the threshold of the Rs to ~15 MOHM and accordingly check whether the data allow the same conclusions.

We thank the reviewer for pointing this out. Indeed, the whole-cell recordings were performed at <30 MOhm Rs, but we compensate it. We clarified it in the revised manuscript (Materials and method section). Following the reviewer’s suggestion, we reduced the threshold of the Rs to ~15 MOhm through using the low resistant recording electrodes (4–6 MOhm). We observed the connection probability and strength of SST-IN=>PC uIPSCs exhibited similar developmental properties (Figure 1—figure supplement 4).

5) It remains unclear why whole-cell recordings have been performed with a K-gluconate based intracellular solution containing ~20 mM Cl^-^. What was the experimentally determined reversal potential of IPSCs? It may have been a value of ~-65 mV (estimate of the reviewer). At a holding potential of -85 mV this will result in a driving force of only ~20 mV. This is a very small driving force for Cl^-^, for the main aim to measure differences in IPSC sizes.

The theoretical reversal potential (calculated by using the Nernst equation) should be ~−50 mV in intracellular solution containing ~20 mM Cl^-^. Therefore, the driving force should be ~35 mV at holding potential of −85 mV. Moreover, the experimentally reversal potential we tested was ~−47.96 ± 1.06 mV (n = 4) under K-gluconate based intracellular solution containing ~20 mM Cl^-^.

In the revised manuscript, we observed the connection probability and strength of SST-IN=>PC uIPSCs exhibited similar developmental properties when we used a Cs-based intracellular solution (improve space clamp) containing high concentration of Cl^-^ (increase the driving force of uIPSCs) to record the postsynaptic currents (Figure 1—figure supplement 4).

6) Figure 4C and D show that the mean difference in the IPSC size between SST-PC connections at P12/13 and at P14/15 depends on few paired recordings in which the IPSC size at P12/13 was above 50 pA (n = 2 pairs). Please provide data on the distribution of IPSC sizes in both IPSC groups.

We have provided data on the distribution of IPSC sizes in Figure 1F as well as in other figures.

7) Increase in connectivity rises already at P9/11 prior to the decline in IPSC amplitude and reduced number of release sites at P14/15 suggesting that morphological growth and synapse function are here two independent processes. This should be emphasized and discussed in the manuscript.

We emphasize and discuss this in the revised manuscript (Discussion section).

8) The here shown data should be discussed in more detail with findings of Miao et al., 2016 who performed at least partly overlapping investigations in the visual cortex during postnatal development. In their study, reduced IPSC size correlated with reduced release sites and increased failure rate.

As mentioned above, Miao et al. analyzed the number of release sites and failure rate in PC=>SST-IN excitatory synapses, whereas we studied SST-IN=>PC inhibitory synapses. We further discussed the difference between Miao et al. and our data in the revised manuscript (Discussion section).

Reviewer #2:

The paper by Yu and colleagues is a worthy endeavour that uses multi-cell patch clamp electrophysiology to interrogate developing synaptic connections between somatostatin (SST)-positive interneurons and other neuronal subtypes, notably pyramidal cells, in the superficial layers of visual cortex. Such electrophysiology investigations are the 'gold standard' as they can provide high-resolution temporal assessment of synaptic kinetics.

1) The Introduction provides a reasonable summary of the field but the references cited could and should be updated to give the reader a more comprehensive and current view of the field. The following are just a few examples:

(i) In the first paragraph, it would apt to cite a number of recent papers on the subject of visual refinement post-eye opening, for example, Hoy & Niell, (2015) and work from the Mrsic-Flogel and Hoffer labs among many.

(ii) The authors state that our knowledge of how eye opening influences inhibitory neurotransmission is limited. However, it would be worth mentioning the work of Kuhlman et al., (2011) who investigated refinement of orientation bias in parvalbumin interneurons as an example of studies that have been performed to date.

(iii) The list of references cited here should be expanded: (i) include reference to Marques-Smith et al., (2016), who identified an early SST interneuron circuits in somatosensory cortex that forms a transient feed-forward GABAergic connections and (ii) include Oh et al., (2016) who demonstrated that GABA release from SST interneurons can promote synaptogenesis in mouse cortex.

We apologize for not citing the referred literatures and added it in revision.

Following these suggestions the authors might like to reconsider the statement and references cited in the Discussion section that "the changes and plasticity [of GABAergic circuits] during eye opening… has generally been neglected".

We revised accordingly.

2) In the Results section: On a number of occasions the authors make vague reference to data that should be included to fully support their conclusions:

(i) I am uncomfortable with the declaration that the authors "explicitly" identified SST interneurons using the Sst-ires-Cre mouse in the absence of further data supporting this claim. There is evidence (Hu et al., 2013) that this GM mouse line labels and can be used to record fast spiking interneurons – typically associated with PV-expressing, basket cells. The authors state that cells were based on morphological and intrinsic electrophysiological data – it is important that this is shown in full.

We thank the reviewer for this valuable point. We systematically analyzed the morphological and electrophysiological properties of tdTomato^+^ neurons in SST-tdTomato mice. We observed ~14.1% of tdTomato^+^ neurons showed the fast-spiking properties (Figure 1—figure supplement 1C). TdTomato^+^ SST-INs were further characterized by morphological properties. After removing these fasting-spiking tdTomato^+^ neurons, we observed the majority of tdTomato^+^ cells in cortical layer 2/3 of the neocortex are the Martinotti cell subtype (95%, 19 out of 20) (Figure 1—figure supplement 1A).

(ii) Provide similar full data sets for the pyramidal cells.

We have provided full data sets for the pyramidal cells accordingly (Figure 1—figure supplement 3).

3) Results section: The authors compare P12-P13 with P14-P15 (see for example Figure 1F,G). Please could they include the other time points recorded to give a more complete picture of the early IPSC kinetics? Comparison of these time points seems odd given the variability in eye-opening reported at P14 (Figure 1—figure supplement 2).

In the revision, we provided the analyzation of uIPSC kinetics at all time points (Figure 1G and 1H).

4) Results section: I am intrigued by the data from cingulate cortex. This is largely ignored in the rest of the manuscript – apart from a brief mention in the discussion – but would appear to suggest that the change in the SST-IN to PC synapse is time locked across all cortical areas and NOT related to eye opening? I appreciate that the subsequent data – for example the eye suturing experiments – support the eye opening-dependent hypothesis but perhaps there are other factors not considered?

We thank the reviewer for this valuable point. During revisions, we found the weakening of SST-IN=>PC synaptic transmission in cingulate cortex could be prevented by binocular lid suture and reproduced by artificial opening of sutured lids (Figure 6D and 6E).

For completeness it would have been good to study PV+ cells to examine whether or not the failure to reduce the amplitude of the SST-PC synapse could be compensating for reduced activity-dependent recruitment of this class of interneuron. I appreciate that the authors suggest that these cells do not change after P9 (Discussion section) but these data are obtained from L5 visual cortex and prefrontal cortex. The argument put forward in the Discussion section for not recording PV+ cells is weak: The Hestrin lab and others have taken advantage of the G42 line to reliably identify putative-PV+ cells in neonatal cortex. A genetic strategy similar to that described on p.15 could have been employed. Indeed, it was likely that a large proportion of the cells labelled with GFP in the GAD-GFP line would have been putative PV+ cells. It is worth noting that Anastasiades et al., 2016 have shown that putative PV+, FS interneurons can be reliably differentiated from SST+, non-FS cells at early neonatal ages on the basis of a combined morphological and electrophysiological assessment.

We appreciate these comments. We have studied the development of synaptic transmission from fast-spiking PV interneurons (FS-INs) to pyramidal cells (PCs) in layer 2/3 of the visual cortex during the time of eye opening. We took advantage of Lhx6-EGFP transgenic mice, in which the majority of MGE-derived interneurons are labeled, and bred this line onto SST-tdTomato line (*SST-tdTomato::Lhx6-EGFP* line), allowing us to distinguish between SST-INs (tdTomato^+^) and other types interneurons (EGFP^+^/tdTomato^-^) (Figure 3A). EGFP^+^/tdTomato^-^ PV-INs were further determined with the fast-spiking properties. Interestingly, unlike synaptic transmission from SST interneurons (SST-INs) to PCs, the strength of FS-IN=>PC synaptic inputs in layer 2/3 of visual cortex was rapidly increased by ~140% from P12–13 (before eye opening) to P14–15 (after eye opening) (Figure 3E). More importantly, we further demonstrated that the dramatic change can be prevented by binocular lid suture and reproduced by artificial opening of sutured lids (Figure 6F and 6G).

5) Results section: The authors should acknowledge that the Htr3a line encompasses a much more diverse population than that studied by Pfeffer et al., (2013). It is important to acknowledge interneuron diversity and not assume that any of the GM lines used provide a homogeneous cohort in the absence of detailed characterization.

We discussed the interneuron diversity in Htr3a line in the revised manuscript (Materials and methods section).

6) Results section: This data could be reasonably condensed or the rationale explained more clearly for why they recorded and separately report data from layer 1, then 2/3 then all the neuronal subtypes together.

We appreciate these comments. We removed layer 1 data in the revision. Instead, the development of synaptic transmission from SST-INs to PV-INs at layer 2/3 of visual cortex has been explored (Figure 4A, 4B and 4C). We found that the connection probability and strength of SST-IN=>FS-IN uIPSCs did not change from P12–13 to P14–15 (Figure 4B and 4C). Moreover, the connection probability and strength of SST-IN=>FS-IN synaptic transmission were comparable at P12–13 and P14–15 in sutured mice (Figure 6—figure supplement 3).

7) Discussion section: This statement about eye-opening and development of other brain regions is not supported by the data presented in the current manuscript.

We omitted this statement.

Reviewer #3:

In this manuscript, Guan and colleagues report an interesting observation, namely that SST-positive interneurons of mouse visual cortical layer 2/3 scale down their inhibitory synaptic responses, following eye opening and selectively on pyramidal neurons. The authors go on and show that this is not due to the actual intrinsic development of cortical circuits, but, rather, it depends on visual sensory activity. They conclude that this effect depends on a combination of pre- and post-synaptic mechanisms at GABAergic synapses between SST interneurons and layer 2/3 pyramidal neurons.

The manuscript is very well written, and the experiments are illustrated with a clear logic. The finding seems robust and quite interesting. The paper has, however, some limitations.

1) Use of Sst-IRES-Cre mouse: whereas this mouse line has been widely used to study SST interneurons in cortical layer 2/3 both in vivo and in vitro, cortical SST cells are heterogeneous (Scheyltjens and Arckens, 2016; Hu et al., 2013; Rossier et al., 2015). The authors claim that they restricted their analysis on Martinotti cells, but the evidence that they provide is anecdotal. Indeed, Figure 1—figure supplement 1 shows only one reconstructed neuron: It would be useful to know if that interneuron was representative of the entire sampled population. The authors should include a quantitative morphological analysis of axonal projections on some recorded neurons to corroborate their claim that they are recording Martinotti cells. Likewise, there is no quantification of the firing properties, but only two representative traces are shown (Figure 1—figure supplement 1B). If the authors want to make the point that they consistently record from Martinotti cells, they should provide more solid evidence.

We thank the reviewer for this valuable point. We systematically analyzed the morphological and electrophysiological properties of tdTomato^+^ neurons in SST-tdTomato mice (Figure 1—figure supplement 1). Indeed, we observed ~14.1% of tdTomato^+^ neurons showed the fast-spiking properties, and exhibited basket-like morphology (Figure 1—figure supplement 1C). After removing these fasting-spiking tdTomato^+^ neurons, we observed the majority of tdTomato^+^ cells in cortical layer 2/3 of the neocortex were the Martinotti cell subtype (95%, 19 out of 20) (Figure 1—figure supplement 1A).

2) The reduction of SST-P GABAergic synapses induced by eye opening seems to be target specific. This is indeed interesting, and the causal link between eye opening and synaptic weakening, is the core of the article. Importantly, however, potential non-specific effects induced by their manipulations should be ruled out. Target specificity of dark rearing and artificial eye opening at P17 is missing: it would be nice to see that dark rearing and/or eye surgery does not affect the other connections (SST-IN => L1-IN or SST-IN => L2/3 Htr3a-IN) as they showed for the developmental characterization.

Thanks for the reviewer’s comments. In the revision, we investigated the development of synaptic transmission from SST-INs to FS-INs during the time of eye opening (Figure 4A, 4B and 4C). The connection probability and strength of SST-IN=>FS-IN synaptic transmission did not change from P12–13 to P14–15 (Figure 4B and 4C). In addition, we found that both the connection probability and strength of SST-IN=>FS-IN synaptic transmission were comparable at P12–13 and P14–15 in sutured mice (Figure 6—figure supplement 3C and 3D).

3) It is not entirely clear to me why the authors chose layer 1 interneurons and 5-HT3a positive cells as other postsynaptic targets. For example, PV and VIP cells are also known to be connected by SST interneurons (Ma et al., 2012; Pfeffer et al., 2013).

We appreciate the comments. The layer 1 data were removed in the revised manuscript. Instead, as mentioned above, we studied the development of SST-IN=>FS-IN synaptic transmission.

4) The data shown in Figure 5 are not completely convincing. Indeed, a proper multiple-probability fluctuation analysis requires changes of extracellular Ca/Mg to estimate quantal synaptic parameters. The fit with a parabolic function of the data shown in Figure 5F is unconvincing, as all points cluster on the left side of the vertex. Moreover, how did the authors exclude receptor saturation and/or desensitization? This is especially problematic for synapses that are far from the recording sites (as is the case of distal dendritic synapses). Moreover, the authors recorded distant synapses using a K-based intracellular solution with relatively low intracellular chloride (~20 mM).

Thanks for reviewer’s valuable comments. In the revised manuscript, we used the theoretically expected parabolic relationship between the variance and mean of synaptic responses under multiple-pulse stimulation and different external Ca^2+^/Mg^2+^ concentrations to obtain estimates of the presynaptic release sites (N) and the postsynaptic quantal size (Q) (Figure 7). Moreover, we used a Cs-based intracellular solution (improve space clamp) containing high concentration of Cl^-^ (increase the driving force of uIPSCs) to record the postsynaptic currents. We found that N at P12–13 and P14–15 were not significantly different in both SST=>PC and FS=>PC synaptic transmission (Figure 7G and 7O). However, Q of SST=>PC synaptic transmission at P14–15 was significantly lower than that at P12–13 (Figure X); conversely, we found a significant increase in Q at P12–13 compared with the mice at P14–15 in FS=>PC connections (Figure 7H and 7P).

5) Effect of the alpha5-IA on synapses between SST-INs and P neurons: Interestingly, there is only a 10% effect of the drug after eye opening. This result does not fit with the data shown by Ali and Thomson, (2008), who recorded from rats after eye opening (P18-22). How do the authors explain these apparent contradicting results?

Given that the precise mechanism underlying the changes of SST-INs and FS-INs onto PCs during eye opening still remains largely elusive, we omitted α5-GABA_A_Rs-related data in the revised manuscript. Instead, we have systematically studied the development of FS-INs=>PC synaptic transmission in layer 2/3 of the visual cortex (Figure 3).

6) The results on cingulate cortex are somehow confusing, as in principle, this cortex does not receive direct visual information, and the authors' interpretation of their results is indeed a visual-mediated effect. Perhaps, the results on cingulate cortex should be either better discussed or removed.

We appreciate the comments. Regarding the concern as to whether eye opening can modulate SST-IN=>PC synaptic transmission in the cingulate cortex, we demonstrated that the weakening of synaptic transmission from SST-INs to PCs in cingulate cortex can be prevented by binocular lid suture, and reproduced by artificial opening of sutured lids (Figure 6D and 6E). Our data suggested that natural eye opening not only affects the maturation of the visual system but also contributes to the proper development of cingulate cortex. Indeed, eye opening has been shown to affect hippocampal development (Dumas et al., 2004).

[Editors’ note: the author responses to the re-review follow.]

Essential revisions:

1) The new data with PV cells are important and add significant impact to the manuscript. The authors show that the developmental change of inhibitory strength from PV cells goes in the opposite direction than that of SST interneurons. The target-specific effects of SST-cell synapses are interesting and this raises the question of whether also PV-PV connections are not affected as PV-PC synapses. Therefore, please perform additional PV-PV paired recordings.

We thank the reviewers for raising this issue. In the revised manuscript, we studied the development of synaptic transmission from fast-spiking PV interneurons (FS-INs) to FS-INs in layer 2/3 of the visual cortex. We found that the connection probability and strength of FS-IN=>FS-IN uIPSCs exhibited no significant difference between the P12–13, P14–15 and P16–19 groups (Figure 4—figure supplement 1). Moreover, both the connection of probability and strength of FS-IN=>FS-IN synaptic transmission exhibited similar results between P12–13 and P14–15 in sutured mice (Figure 6—figure supplement 4).

In addition, we systematically studied the development of FS-IN=>PC synaptic transmission in layer 2/3 of Cg1/2. Similar to V1, we observed the peak amplitude of FS-IN=>PC uIPSCs at P14–15, and P17–20 was significantly higher than that at P12–13 (Figure 3—figure supplement 2), while the connection probability remained comparable among P12–13, P14–15 and P17–20 (Figure 3—figure supplement 2). Importantly, we demonstrated that strengthening of FS-IN=>PC transmission in layer 2/3 of Cg1/2 can be prevented by binocular lid suture, and reproduced by artificial opening of sutured lids (Figure 6I). Furthermore, no significant changes in the connection probability were observed betweeen P12–13, P14–15 and P17–20 sutured mice, and P17–20 artificially opened mice (Figure 6H).

2) It remains unclear what factor induces the change in synaptic inhibitory strength. Since it is a postsynaptic effect, direct signaling mechanisms induced by the presynaptic interneuron are not likely to be involved (failure rate, release probability are unchanged). Since the main observation was obtained in both the visual and cingulate cortex, it remains unclear whether the here described main phenomenon has indeed something to do with light-mediated factors (vision in general) or vision-related factors such as mobility directly or indirectly induced by the siblings or mother. Therefore, please discuss more the effect of eye opening in cortical brain regions despite the visual cortex.

Thanks for reviewer’s comments. We discuss the vision-related factors in the revised manuscript (Discussion section).

3) Improve the spelling.

We have carefully revised the spelling throughout the manuscript.

4) Formulate a hypothesis what factor may cause the postsynaptic mechanisms supporting increase in the quantal content.

We discussed the postsynaptic mechanisms underlying increase/decrease in the quantal content (Discussion section). Since GABA_A_ receptor subunit expression in neocortex exhibits significant changes during early postnatal development (Bosman, et al., 2002; Fritschy, et al., 1994; Heinen, et al., 2004), our main hypothesis is that the changes in subunit expression and/or composition of the GABA_A_ receptor may induce the differential developmental changes of SST-IN=>PC and FS-IN=>PC synaptic strength during eye opening.
